# Ginseng-mulberry (medicine-food homologous) pair mitigates cadmium-induced anxiety: a clinical proteomics-guided network pharmacology with rat validation

**DOI:** 10.3389/fpsyt.2026.1792233

**Published:** 2026-05-25

**Authors:** Maoqin Tian, Sheng Wan, Hao Gao, Yinghui Yin, Feng Han, Yong Yang, Zhenzhong Liu, Qihan Zhao, Shaoxin Huang

**Affiliations:** 1 School of Public Health, North Sichuan Medical College, Nanchong, China; 2 Jiangxi Provincial Key Laboratory of Cell Precision Therapy, School of Basic Medical Sciences, Jiujiang University, Jiujiang, Jiangxi, China; 3 Jiangxi Provincial Key Laboratory of Disease Prevention and Public Health, Nanchang, Jiangxi, China; 4 Jiujiang University Clinical Medical College, Jiujiang University Hospital, Jiujiang, China; 5 SpecAlly Life Technology Co., Ltd., Wuhan, Hubei, China; 6 Jiangxi Jiujieli Life Technology Co., Ltd., Jiujiang, Jiangxi, China

**Keywords:** anxiety, cadmium, clinical proteomics, ginseng, mulberry leaf, network pharmacology

## Abstract

**Background:**

Cadmium(Cd) exposure is associated with anxiety, but mechanism-informed multi-target interventions are limited.

**Methods:**

We profiled plasma proteomes by LC-MS/MS in a propensity-score-matched Cd-exposed cohort (25 anxious vs 25 non-anxious). Anxiety-associated proteins were used as seeds for reverse screening in TCMIP v2.0, followed by network integration, molecular docking, 100ns molecular dynamics simulations, and rat validation of a ginseng-mulberry leaf (medicine-food homologous) decoction.

**Results:**

Proteomics identified 120 differentially expressed proteins associated with anxiety. Network screening prioritized ginseng and mulberry leaf; quercetin and kaempferol showed strong and stable binding to AKT1, PTGS2, and HSP90AA1. In Cd-exposed rats, the decoction increased open-field center exploration without altering locomotion, attenuated prefrontal perivascular enlargement and glial dysmorphology, and restored AKT1/PTGS2 immunofluorescence.

**Conclusions:**

A clinical proteomics-initiated discovery pipeline suggests a poly-pharmacological intervention for Cd-induced neurotoxicity. The ginseng-mulberry leaf pair counteracts Cd-associated neurobehavioral and prefrontal changes and warrants dose-response and target-perturbation studies.

## Highlights

- Propensity-score-matched clinical proteomics identifies a cadmium-associated anxiety signature (120 DEPs).
- Proteomics-seeded network pharmacology nominates a ginseng-mulberry leaf pair and core nodes (AKT1/PTGS2/HSP90AA1).
- The decoction reverses Cd-induced anxiety-like behavior and prefrontal neurovascular/glial pathology in rats.

## 1 Introduction

Chronic low-dose environmental cadmium (Cd) exposure has been epidemiologically linked to anxiety, but mechanism-informed, multi-target interventions remain limited in environmental toxicology. Anxiety disorders affect hundreds of millions of people worldwide (1, 2), and environmental pollutants are increasingly recognized as modifiable risk factors. Experimental evidence indicates that Cd can disrupt the central nervous system through oxidative stress, mitochondrial impairment, and neurotransmitter imbalance (3, 4). Current pharmacotherapies may be constrained by incomplete response and adverse effects (5, 6). Traditional Chinese medicine (TCM), with multi-component and multi-target properties, offers a complementary strategy for complex exposure-related phenotypes (6, 7). For example, curcumin can chelate Cd and modulate oxidative and inflammatory pathways to attenuate anxiety-like behaviors (6).

Most cadmium neurotoxicity studies rely on empirical candidate testing and single-pathway hypotheses. Network pharmacology can integrate multi-target mechanisms, yet many applications focus on canonical anxiety models and are less tailored to environmental toxicology (3, 7). Integrating network pharmacology with exposure-linked omics (e.g., proteomics) can improve biological relevance and reduce the hypothesis space (8). Here, we develop a clinical proteome-initiated TCM discovery (CP-ID) framework that couples human plasma proteomics with systems network pharmacology and *in vivo* validation to nominate multi-target interventions for cadmium-associated anxiety.

Although network pharmacology methods can be employed for the screening and prediction of traditional Chinese medicine, the inherent complexity in network construction and analysis may introduce errors in interaction predictions, potentially affecting the accuracy of the results (9). Molecular docking and molecular dynamics can simulate atomic-level interactions between small molecule compounds and protein targets, predict ligand and receptor conformations, and calculate affinities to evaluate combinations (10). These computational chemistry approaches are well established for drug design and the elucidation of biochemical pathways (11).

Briefly, we (i) profiled plasma proteomes by LC-MS/MS in a propensity-score-matched Cd-exposed cohort to predict anxiety-associated proteins; (ii) used these proteins as seeds for reverse screening in TCMIP v2.0 to prioritize herbs and components; (iii) integrated network analysis, molecular docking, and molecular dynamics to refine core compounds/targets; and (iv) validated a ginseng-mulberry leaf decoction in a rat Cd model.

## 2 Materials and methods

### 2.1 Human cohort: screening, eligibility, anxiety ascertainment, and proteomics sampling

**Figure 1:**
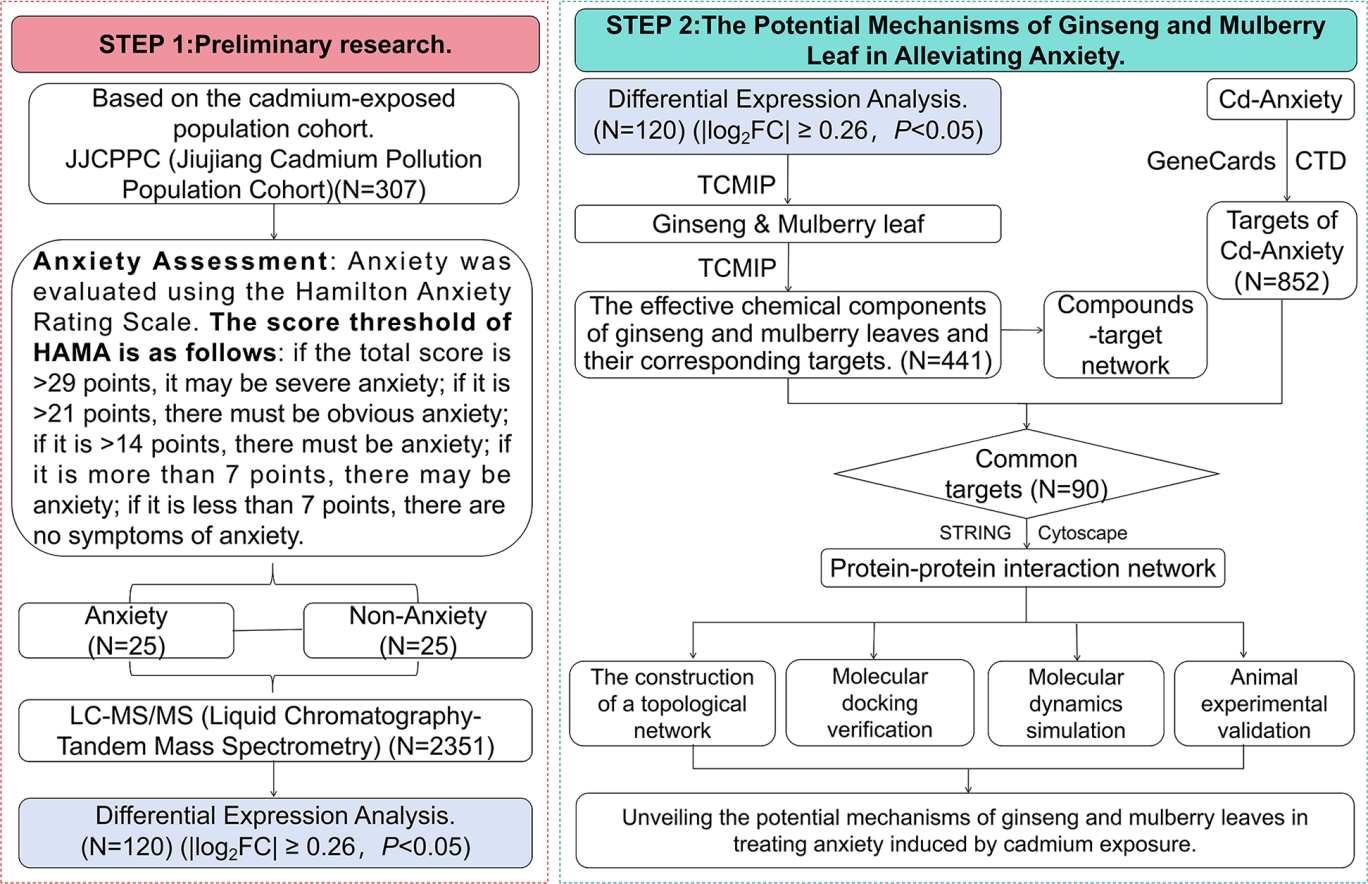
CP-ID framework flow chart. The left section involves identifying proteomic signatures from the cohort. The right section focuses on exploring potential mechanisms, including screening traditional Chinese medicines, constructing networks, molecular docking, molecular dynamics simulations and animal experiments.

This study was conducted in strict accordance with the Declaration of Helsinki and was approved by the Population Research Ethics Committee of Jiujiang University (Approval No. JJVM20240008). All participants provided written informed consent. Participants were recruited from the Jiujiang Cadmium-Contaminated Population Cohort (JJCPPC). Screening and enrollment are summarized in **Figure 1**. Of 307 adults screened in the cohort, 198 met prespecified eligibility criteria (details in **Supplementary Methods**). Anxiety status was ascertained using the 14-item Hamilton Anxiety Rating Scale (HAMA-14); participants with HAMA-14 ≥7 were classified as anxious (ANX) and those with HAMA-14 <7 as non-anxious (NON-ANX) (12). G*Power(v3.1.9.7) was used for *a priori* sample size determination in this discovery proteomics study. Based on the prespecified assumptions, a significance level of α= 0.05, a target statistical power of 1-β = 0.80, and an anticipated protein abundance effect size of 2-fold, our finalized cohort of 50 participants fully satisfies this pre-calculated threshold and aligns with established methodological requirements for high-throughput clinical proteomics. To ensure covariate balance for proteomics analyses under a case-control framework, we implemented 1:1 propensity score matching (PSM) on anxiety status, yielding a matched analytic set of 25 ANX and 25 NON-ANX participants (n = 50). Matching factors were sex (exact), body-mass index (BMI; within ± 10%), age (± 10%), and years of education (± 3 years), consistent with our prespecified protocol. Post-matching balance was confirmed by standardized mean differences < 0.1 for all matching covariates. Clinical characteristics of the two groups were compared according to the cross-sectional design (13).

Peripheral blood was collected under fasting conditions. Plasma proteomes were profiled by LC-MS/MS as detailed in the **Supplementary Methods**. In total, 2,531 proteins were quantified. Differential expression between ANX and NON-ANX groups was assessed at |log2 fold-change| ≥ 0.26 with P < 0.05, yielding 120 differentially expressed proteins (DEPs) that seeded subsequent network pharmacology analyses. These 120 DEPs are considered to be associated with environmental cadmium exposure and anxiety, and are used for the exploratory screening of candidate Chinese herbal medicines. To examine exposure-response relationships, we evaluated correlations between whole-blood cadmium concentrations and HAMA-14 scores, and fitted multivariable logistic regression models for odds of anxiety across Cd exposure (covariates as above), reporting odds ratios (ORs) and 95% *CI* (13).

### 2.2 Network pharmacology-based screening for candidate herbs

To identify potential Chinese medicines for mitigating Cd-induced anxiety, a clinically-driven reverse screening strategy was implemented. The 120 DEPs identified from the clinical plasma proteomics (Section 2.1) served as input seeds in the Traditional Chinese Medicine Integrated Pharmacology Research Platform v2.0 (TCMIP, http://www.tcmip.cn/) (14). By mapping these DEPs onto the “Herb-Target” association modules, TCM with the highest target-hit enrichment scores were prioritized. Based on this enrichment and its traditional use in neuro-protection, the top TCM was selected as the primary candidate for further investigation. Chemical profiling of the selected herbs was conducted using the “Chinese Medicinal Materials Database” within TCMIP. To ensure pharmacological relevance, bioactive components were filtered using the Quantitative Estimate of Drug-likeness (QED) metric. Only compounds exhibiting moderate-to-good drug-likeness (QED≥0.49) were retained for further analysis (14). By using the “Chinese Medicinal Materials Database”, the targets corresponding to the retained compounds were integrated, and after removing duplicate targets, the targets of candidate TCM were obtained.

### 2.3 Disease targets acquisition and intersection analysis

The disease targets were identified through GeneCards (GeneCards: https://www.genecards.org/) and the Comparative Toxicogenomics Database (CTD: http://ctdbase.org/). Search keywords included “Cadmium”, “Anxiety” and “anxiety disorder”. By integrating the targets from two databases and removing duplicates, a comprehensive list of relevant targets was obtained. To identify the core therapeutic targets of candidate TCM against Cd-induced anxiety, a Venn diagram analysis was performed to determine the intersection between the herb-related targets and the disease-related targets. These overlapping proteins were defined as potential pharmacological targets.

### 2.4 GO/KEGG and target organ network analysis

To further elucidate the biological mechanisms, we performed Gene Ontology (GO) and Kyoto Encyclopedia of Genes and Genomes (KEGG) pathway enrichment analyses using Metascape (http://metascape.org/). Furthermore, we analyzed the tissue-specific distribution of core targets via the BioGPS database (similarity threshold = 0.9) to build a “target-organ” network. This spatial perspective allowed us to identify the key action sites of the TCM in mitigating Cd-induced toxicity.

### 2.5 Dual-network integration strategy for core predicted target identification

To pinpoint the most robust therapeutic targets, a dual-network screening strategy was implemented to identify nodes that bridge biological significance and pharmacological relevance. Firstly, the 90 common targets were subjected to Protein-Protein Interaction (PPI) analysis. The CytoHubba plugin was utilized to evaluate nodal importance via two distinct algorithms: Degree, which prioritizes targets with high connectivity, and Maximal Clique Centrality (MCC), which identifies essential structural proteins within dense sub-networks. Concurrently, a “Drug-Component-Target-Pathway” network was constructed using Cytoscape (v3.10.0) to elucidate the systemic interactions and regulatory landscapes of the TCM constituents. To prioritize exploratory candidates that potentially possess both biological centrality and pharmacological accessibility, we extracted the intersection of the top 22 hub nodes from the PPI network and the top 20 targets ranked by degree in the Drug-Component-Target-Pathway network. These overlapping proteins were designated as the core therapeutic targets for subsequent molecular docking and experimental validation.

### 2.6 Molecular docking and molecular dynamics simulation

To evaluate the binding affinity and dynamic stability between core bioactive components and hub targets, we performed in silico verification integrating molecular docking and molecular dynamics simulations. Specifically, the 3D structures of core targets were retrieved from the RCSB Protein Data Bank (PDB, https://www.rcsb.org/), while the molecular structures of bioactive components were obtained in mol2 format from the TCMSP database. Molecular docking was conducted using the CB-Dock2 server, which implemented a blind docking strategy. The server utilized the Curvature-based Cavity Detection (CCD) algorithm to automatically identify potential binding pockets and employed AutoDock Vina to calculate the binding affinities (15). The docking pose with the lowest Vina score was selected as the optimal conformation and visualized using PyMOL (v2.5). Subsequently, to validate the stability of these complexes under dynamic physiological conditions, 100 ns MD simulations were performed using GROMACS (v2021.3) with the AMBER99SB-ILDN force field. The complexes were solvated in a dodecahedron box with TIP3P water molecules and neutralized with Na^+^ or Cl^-^ ions. After energy minimization and equilibration under NVT and NPT ensembles at 310K, a 100 ns production run was executed. The structural stability was assessed via root mean square deviation (RMSD) and root mean square fluctuation (RMSF).

To quantitatively evaluate the binding affinities of the predicted ligand-target complexes, the Molecular Mechanics Poisson-Boltzmann Surface Area (MM-PBSA) method was applied using the gmx_MMPBSA package. To ensure thermodynamic equilibration, snapshots were extracted at regular intervals (every 20 frames) exclusively from the final 20 ns of the 100 ns MD trajectories. The calculations were performed using the AMBER99SB-ILDN force field. The polar solvation free energy was calculated using the Generalized Born (GB) implicit solvent model (igb=5) with a physiological salt concentration of 0.15 M. The total binding free energy (ΔG_bind_) was determined by summing the gas-phase molecular mechanics energy (van der Waals and electrostatic interactions) and the solvation free energy. Furthermore, to explore the micro-environmental binding mechanisms, a per-residue free energy decomposition was performed for all amino acid residues. This allowed for the precise identification of key “hot-spot” residues contributing most significantly to the complex stability.

### 2.7 Establishment of a rat cadmium exposure model, drug intervention, and behavioral testing

SPF Sprague-Dawley rats were obtained from SCBS Biotechnology Holding Co., Ltd. and housed under controlled conditions (25 ± 1°C, 60% humidity, ≤ 60 dB(A), 12-h light-dark cycle) with ad libitum access to food and water. The protocol was approved by the Jiujiang University Medical Ethics Review Committee (approval no.: JJ.No20241111S0540165[012]). Rats were randomized by body weight; behavioral scoring and histology were performed under blinded assessment. Rats were assigned to three groups (n = 6/group): control, cadmium-exposed (Cd), and cadmium-exposed plus ginseng-mulberry leaf decoction (Cd + TCM). Cadmium chloride was administered by oral gavage at 1 mg/kg/day for 8 weeks to establish the model. Subsequently, the intervention group was administered the decoction by gavage (1 mL/kg/day, equivalent to 1.98g crude drug/kg/day, with a ginseng: mulberry leaf ratio of 9:10) for a 4-week intervention period. Decoction preparation followed a two-step aqueous extraction and concentration to 1.98g crude drug/mL; preparations were stored at 4°C and returned to room temperature before dosing (16, 17).

Behavioral tests were conducted at the end of week 12. The Open Field Test (OFT) was used to assess locomotion and anxiety-like behavior. Rats were acclimated for 3–5 days before testing. The chamber (100cm × 100cm × 48cm) was cleaned with 75% ethanol between trials. A fixed camera recorded behavior for 5min, and Smart v3.0 software quantified total distance, center entries, and center duration. Experiments were conducted under consistent lighting with sufficient inter-animal intervals to minimize olfactory cues. Data were analyzed using the Kruskal-Wallis test with Dunn’s *post hoc* comparisons (GraphPad Prism 9.0; SPSS 27.0).

### 2.8 Histopathological analysis and immunofluorescence

To observe the morphological changes in rat brain tissue, the rats were sacrificed at week 13. The rats were euthanized by intraperitoneal injection of an overdose of pentobarbital sodium (at a dose of 200 mg/kg). The brain tissue was removed and fixed in 4% paraformaldehyde (BioProE, B32001B). The frontal lobes of three rats from each group were fixed in paraffin for 24h and embedded. The tissue was then sectioned to a thickness of 5 μm and stained with hematoxylin and eosin (HE). The pathological sections were observed under a microscope to analyze the degree of damage to the frontal lobe tissue. To verify the accuracy of the target sites selected by our computational chemistry screening, immunofluorescence analysis was performed on the rat frontal lobe tissue. The sections were dewaxed, antigen-retrieved, and blocked as required, and then incubated with primary antibodies (AKT1, 1:200, AY0420, Abways; PTGS2, 1:200, 12282S, CST) in antibody diluent at 4°C overnight. After washing with PBS, the sections were incubated with fluorescence-conjugated secondary antibody for 1h at 37°C. Then, the sections were incubated with TSA reagent (AKT1, TYR-488; PTGS2, TYR-555) for 30min at 37°C, followed by washing with PBS. Finally, the sections were stained with DAPI for 5min at room temperature, washed with PBS, and mounted with antifade mounting medium. The stained sections were observed and imaged under a fluorescence microscope.

## 3 Results

### 3.1 Clinical characterization and blood cadmium levels in the propensity score-matched cohort

**Figure 2:**
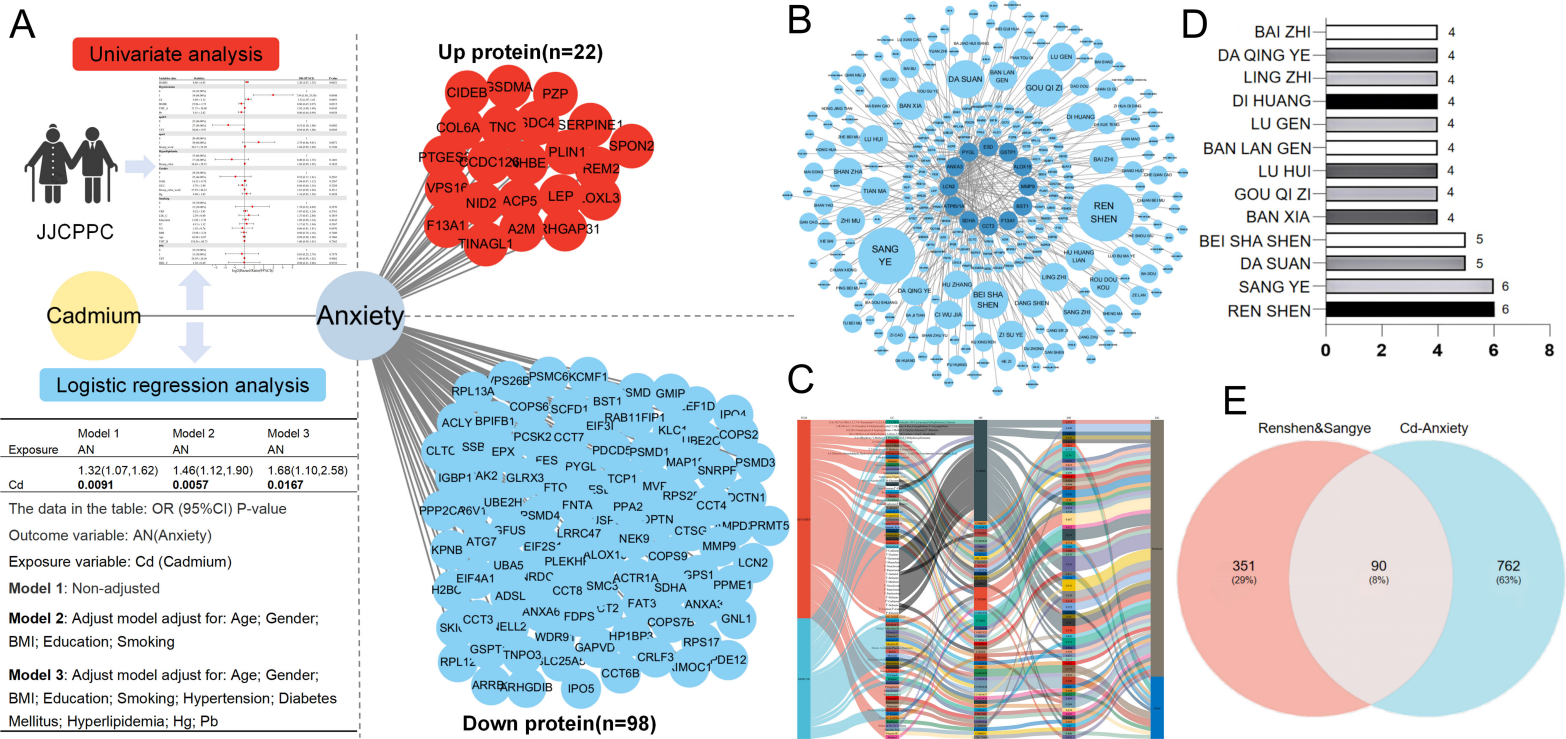
Common targets analysis of ginseng and mulberry leaf for cadmium-induced anxiety. **(A)** Overview of cadmium-linked anxiety targets identified from JJCPPC by plasma proteomics. **(B)** The screening network diagram of traditional Chinese medicine. **(C)** Visualization of the chemical space, drug-likeness (QED), and molecular formulas of the bioactive components from ginseng and mulberry leaf. **(D)** Bar plot ranking the top herbs identified from the reverse screening. **(E)** The venn diagram of 90 common targets between Renshen & Sangye and Cd-Anxiety.

**Supplementary Table 1** presents the baseline characteristics of the participants. Within the matched cohort (n = 50), blood Cd levels were higher in the anxiety group than controls (6.09 ± 3.02 vs. 3.69 ± 2.79 μg/L, *P*= 0.005). Multivariable logistic regression indicated higher Cd exposure was associated with increased odds of anxiety (ORs: 1.32, 1.46, and 1.68; all *P*< 0.05). Participants with anxiety also showed lower MMSE scores (*P*= 0.014) and longer TMT-A times (*P*= 0.024). These clinical findings provided the anchor for the proteomics-derived 120 DEPs (**Figure 2A**) used in the CP-ID framework.

### 3.2 Clinical proteome-initiated discovery of ginseng and mulberry leaf pair and target identification

Reverse screening identified 13 potentially valuable traditional Chinese medicines, including ginseng and pinellia (**Figures 2B, D**). To ensure the safety and translational feasibility of interventions for long-term chronic environmental exposure, the final selection focused on medicine-food homology substances as candidate. Drawing on “Shennong’s Classic of Materia Medica” and “Compendium of Materia Medica”, we selected ginseng and mulberry leaf. They are documented to have the effects of tonifying and calming the spirit, and clearing the liver and brightening the eyes, respectively. Using the QED scores from the TCMIP, we identified 39 components with moderate to good drug-likeness (QED ≥ 0.49). The Sankey diagram illustrates the chemical profiling associations of the ginseng-mulberry pair and its candidate components, revealing the material basis and druglikeness characteristics(chemical composition(CC), molecular formula(MF), drug-likeness weight(DW)) underlying its therapeutic efficacy (**Figure 2C**). By extracting and integrating the corresponding genes of these components, we identified 441 related genes, which were subsequently imported into Cytoscape to construct a network diagram (**Supplementary Figure 1**). The network, comprising two traditional Chinese medicines, 39 chemical components, and 441 targets, revealed that compounds such as adenosine, kaempferol, and quercetin exhibited strong interactions with the targets (the correspondence between TCM and components is detailed in **Supplementary Table 2**). This finding indicated a multi-compound, multi-target mechanism underlying the therapeutic potential of ginseng and mulberry leaf against cadmium-induced anxiety.

### 3.3 Intersection target groups of ginseng and mulberry leaf in anti-cadmium related anxiety screened from multiple databases

Disease targets were sourced from public databases. A search of GeneCards and the Comparative Toxicogenomics Database (CTD) for “cadmium exposure” and “anxiety” returned 844 and 23 targets. These were consolidated into a non-redundant set of 852 potential disease targets. To identify the most relevant therapeutic points, this disease target set was compared with the candidate targets of the drug, revealing 90 common targets (**Figure 2E**; molecular functions are detailed in **Supplementary Table 3**).

### 3.4 GO/KEGG and target organ network analysis revealed common targets converging on neuro-immuno-metabolic function axes

**Figure 3:**
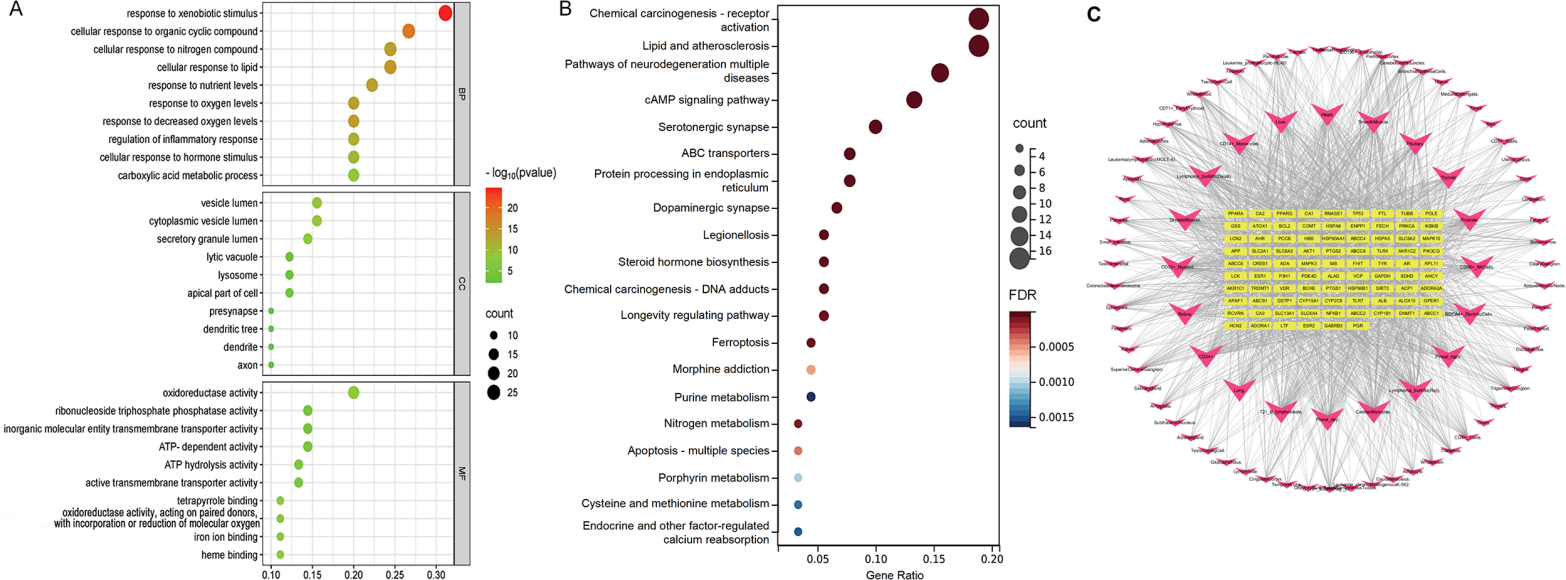
Results of enrichment analysis and organ network. **(A)** Bubble plot of GO functional enrichment analysis. **(B)** Bubble chart of the top 20 pathways based on KEGG enrichment analysis. **(C)** The target organ network analysis.

The Gene Ontology (GO) annotation results showed that the core targets are mainly localized in key neuronal structures such as the “presynapse,” “dendrite,” and “axon” (CC), primarily involving molecular functions and biological processes that include “oxidoreductase activity,” “response to xenobiotic stimulus,” and the regulation of “oxygen levels”. This suggests that the ginseng and mulberry leaf exerts neuroprotection by maintaining synaptic integrity while counteracting Cd-induced oxidative stress and toxicity. Validating this hypothesis, KEGG pathway analysis identified the enrichment of critical neuro-modulatory signaling axes, notably the “cAMP signaling pathway” and “Serotonergic/Dopaminergic synapse” pathways, alongside cell survival mechanisms such as “Ferroptosis” and “Pathways of neurodegeneration”. Furthermore, the target-organ network analysis demonstrated that these validated targets (e.g., AKT1, PTGS2) exhibit tissue-specific high expression in the Prefrontal Cortex (PFC) and Hippocampus, the brain’s centers for emotional regulation, as well as the Liver. Collectively, these findings propose a “neuro-immuno-metabolic” mode of action, wherein the GM pair alleviates anxiety by simultaneously restoring brain synaptic plasticity (via cAMP and monoamine regulation), inhibiting neuronal ferroptosis, and modulating systemic metabolic detoxification (**Figure 3**).

### 3.5 Identification of core targets via dual-network convergence strategy

**Figure 4:**
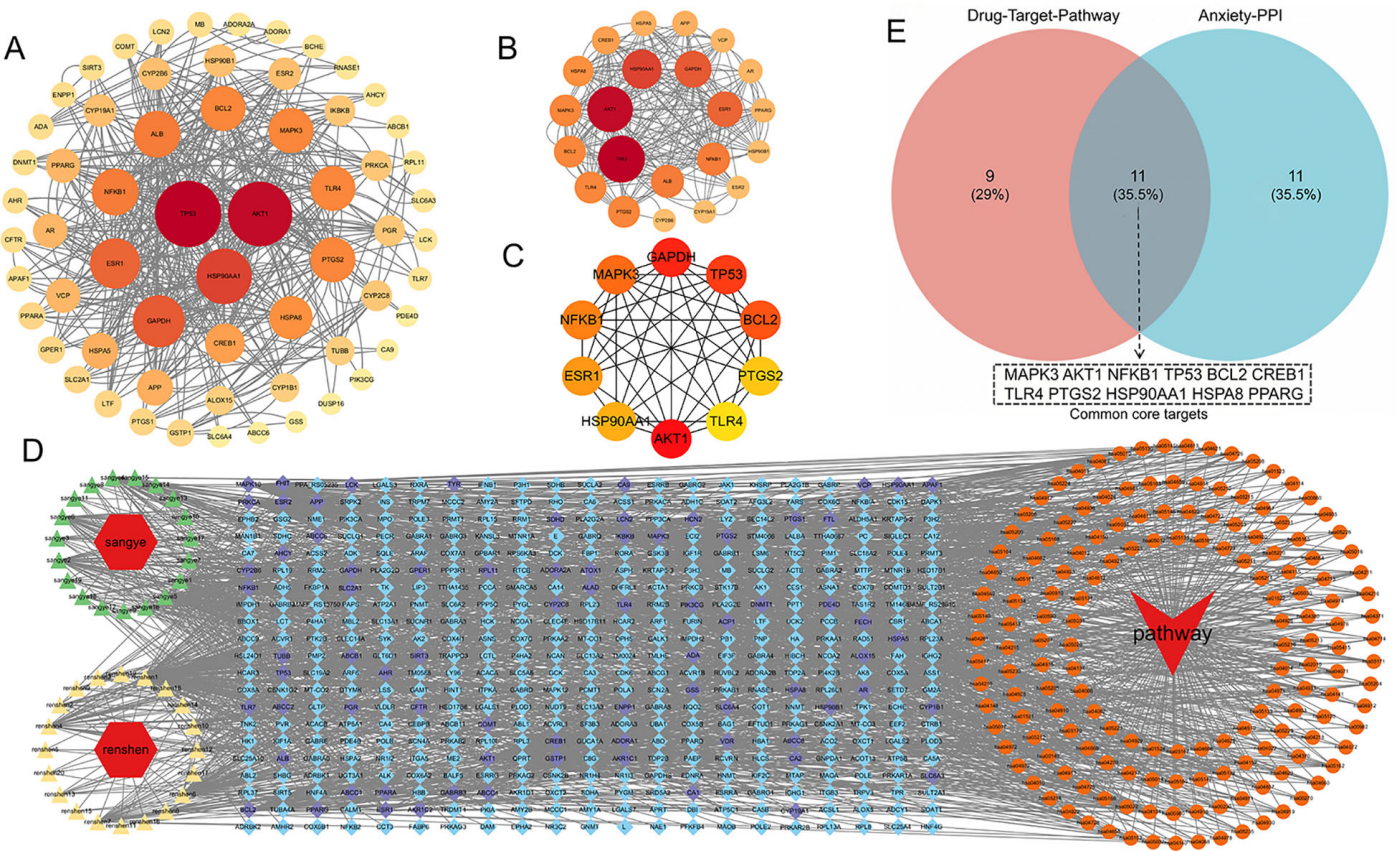
Identification of core targets of the ginseng-mulberry pair against Cd-induced anxiety via a dual-network integration strategy. **(A)** Protein-Protein interaction (PPI) network. **(B)** Hub targets identification by degree. **(C)** Essential clusters identification by MCC. **(D)** Herb-component-target-pathway network. **(E)**Venn diagram of dual-network.

The identification of core therapeutic targets followed a rigorous dual-network convergence strategy (**Figure 4**). The PPI network revealed complex biological interactions (**Figure 4A**). Analysis based on multiple algorithms further identified AKT1, TP53, PTGS2, HSP90AA1, and MAPK3 as key targets exhibiting both high degree and high MCC within the network (**Figures 4B, C**). This topological centrality suggests that these targets serve as primary regulatory nodes through which the ginseng-mulberry pair exerts its holistic anti-anxiety effects, providing a high-confidence reference for downstream validation studies. Simultaneously, to define the pharmacological core, a “Herb-Component-Target-Pathway” network was constructed, comprising 39 bioactive components (e.g., quercetin, kaempferol, and ginsenosides) and their respective targets (**Figure 4D**). From this network, the top 20 pharmacological hubs were prioritized based on their connectivity degree and involvement in anxiety-related signaling pathways (**Table 1**). Furthermore, the top 10 bioactive compounds were identified as the primary material basis (**Table 2**). Finally, the dual-network venn diagram pinpointed 11 candidate targets possessing both biological and pharmacological significance.

**Table 1:** Top 20 targets ranked by degree in the drug-target-pathway network.

Gene name	Degree	Gene name	Degree	Gene name	Degree	Gene name	Degree
MAPK3	109	PRKCA	60	PTGS2	25	SDHD	12
AKT1	98	TP53	48	APAF1	21	PPARG	12
NFKB1	75	BCL2	42	SLC2A1	17	HSP90B1	11
MAPK10	71	CREB1	40	HSP90AA1	16	HSPA8	11
IKBKB	60	TLR4	28	PIK3CG	13	PPARA	11

**Table 2:** Detailed information of the top 10 compounds in the drug-target-network.

Rank	Component name	Structure	Degree	QED
1	Adenosine	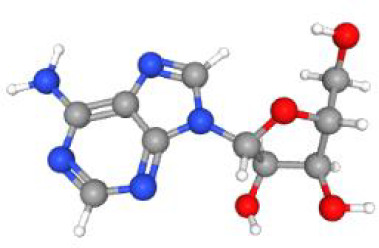	155	0.495
2	Kaempferol	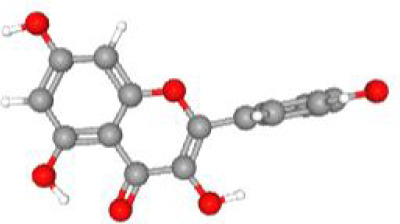	73	0.637
3	Quercetin	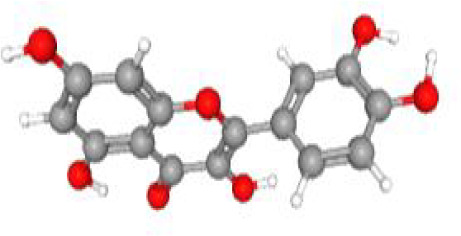	73	0.541
4	Pentanic Acid	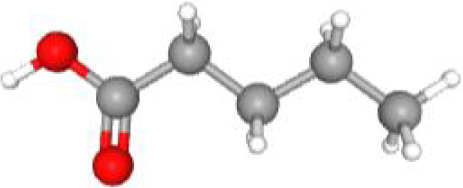	50	0.584
5	5,7-Dihydroxychromone	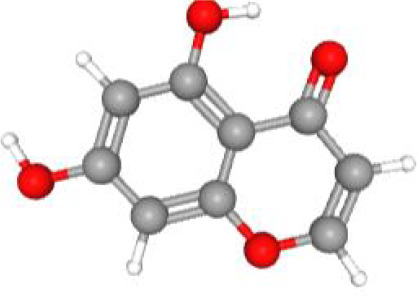	41	0.619
6	Campesterol	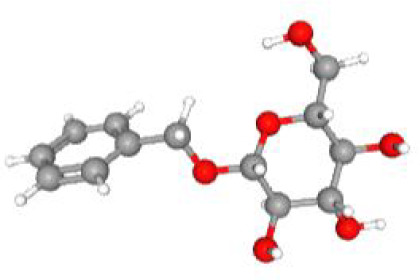	40	0.587
7	Succinic Acid	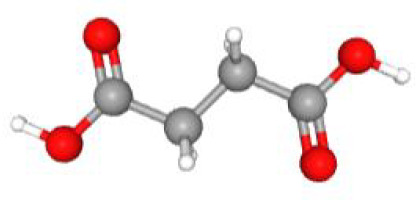	34	0.530
8	Ginsenol	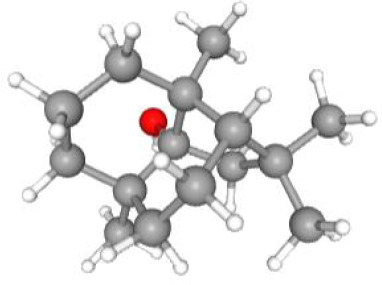	32	0.666
9	Cudranin	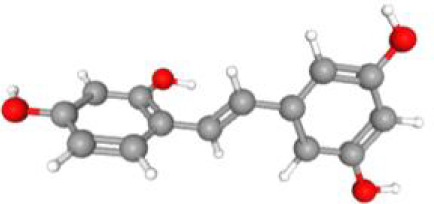	29	0.612
10	Panaxatriol	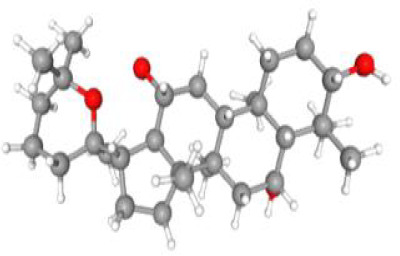	27	0.528

### 3.6 Validation of high-affinity interaction patterns of core target complexes for anti-anxiety effects of ginseng and mulberry leaf based on molecular docking analysis

**Figure 5:**
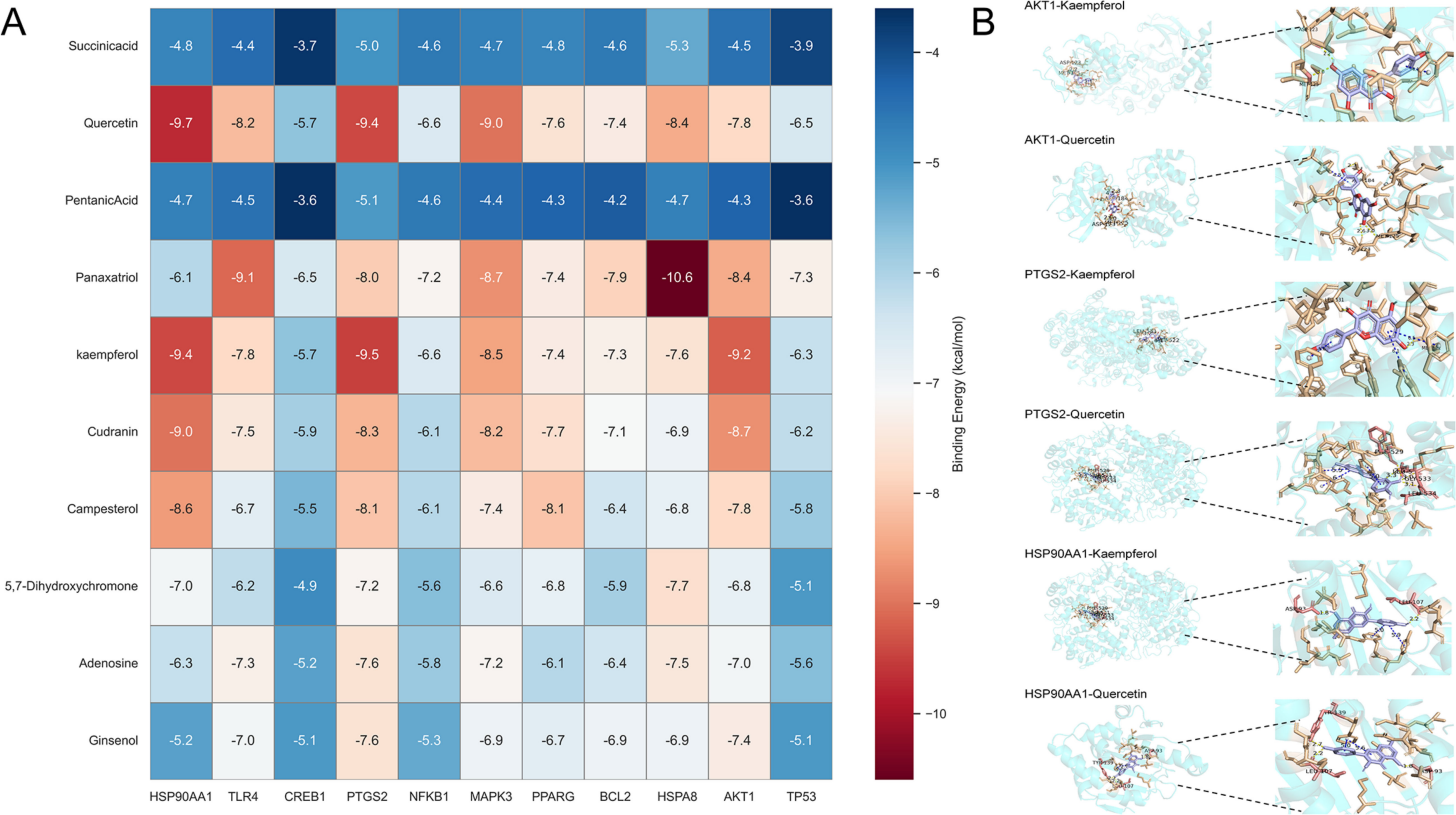
Molecular docking results of core targets. **(A)** Molecular docking binding energy heatmap. **(B)** Binding energies (kcal/mol) of quercetin and kaempferol with their respective targets (HSP90AA1, PTGS2, AKT1).

The CB-Dock2 molecular docking platform was employed to investigate the interactions between 11 common core targets and the top 10 ranked compounds. A total of 110 ligand-receptor pairs, involving 11 core targets (e.g., AKT1, PTGS2, and HSP90AA1) and the top 10 compounds, were analyzed. The receptor structures were retrieved from the RCSB Protein Data Bank (PDB IDs: 4bqg, 3fxi, 5zko, 4qtb, 8tqd, 5f19, 8wfe, 1g5m, 4h5r, 3d06, and 3o96). The docking results, visualized via a binding energy heatmap (**Figure 5A**), demonstrated that all pairs exhibited low binding energies, suggesting geometrically favorable interactions that prioritize these complexes for further dynamic validation. Notably, Quercetin and Kaempferol displayed the most prominent affinities. Through literature research, the core predicted components identified in this screening (e.g., quercetin, kaempferol) have been consistently validated in similar aqueous extracts using high-resolution analytical techniques in previous authoritative studies (**Supplementary Table 4**) (18–23). Structural visualization using PyMOL revealed that these compounds anchored firmly into the active pockets of their respective targets through a combination of hydrogen bonds, π-π, and hydrophobic interactions (**Figure 5B**), suggesting their role as the primary chemical basis for the anti-anxiety effects of the ginseng-mulberry pair.

### 3.7 Structural stability and conformational dynamics via molecular dynamics simulations

To elucidate the binding stability and residue-level flexibility of the core targets (AKT1, HSP90AA1, and PTGS2) with their respective active constituents (kaempferol and quercetin), 100ns molecular dynamics (MD) simulations were performed. The root mean square deviation (RMSD) was calculated to evaluate the structural equilibrium of the complexes. HSP90AA1-kaempferol demonstrated exceptional stability, maintaining a rigid conformation with a remarkably low RMSD of 1.12± 0.08Å. The HSP90AA1-quercetin and PTGS2-quercetin complexes also reached rapid convergence, stabilizing at 2.34± 0.15Å and 1.92± 0.12Å, respectively. In contrast, the PTGS2-kaempferol system exhibited significant structural deviation, with RMSD values escalating to 16.24± 2.45 Å, indicating an unstable binding mode. Conversely, the FEL of PTGS2-kaempferol was highly dispersed and lacked a well-defined energy well, further validating the transient nature of their interaction.

**Figure 6:**
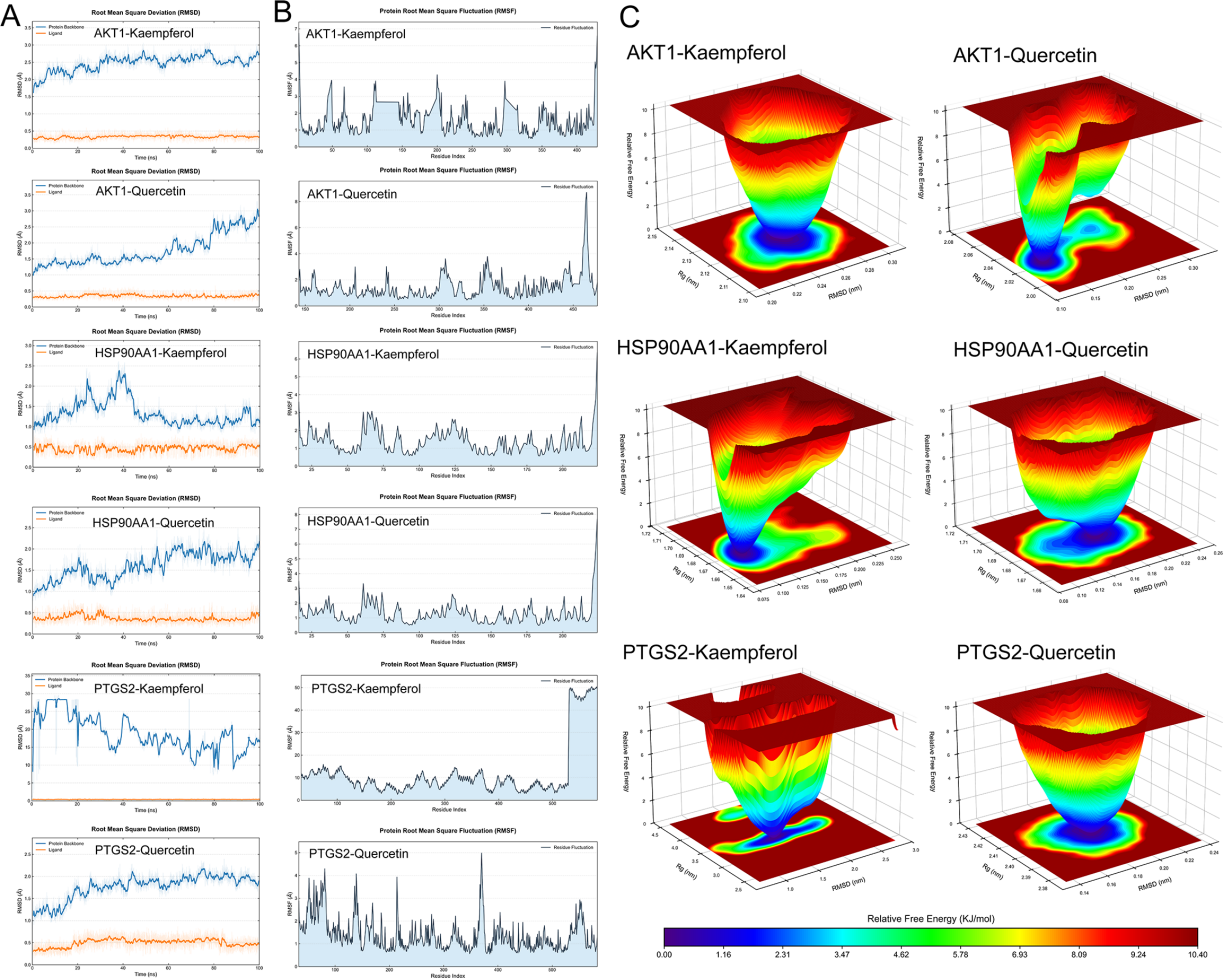
Molecular dynamics simulation parameter time history analysis. (**A**) The RMSD values of protein-ligand complexes over time. **(B)** The RMSF value of the amino acid backbone of the protein-ligand complex over time. **(C)** Protein-Ligand free energy landscape.

MM-PBSA calculations were performed on a dense ensemble of snapshots extracted exclusively from the fully converged phase of the trajectories. The quantitative thermodynamic analysis revealed highly favorable total binding free energies (ΔG_total_) for all screened complexes (**Supplementary Figure 2**). Specifically, Quercetin exhibited exceptionally robust binding affinities across the predicted targets (PTGS2-Quercetin: -28.08 ± 0.80kcal/mol; AKT1-Quercetin: -25.87 ± 0.84kcal/mol; HSP90AA1-Quercetin: -25.75 ± 0.94kcal/mol). Kaempferol also demonstrated stable thermodynamic engagements (HSP90AA1-Kaempferol: -8.70 ± 1.38kcal/mol; PTGS2-Kaempferol: -7.82 ± 1.38 kcal/mol; AKT1-Kaempferol: -6.92 ± 1.42 kcal/mol). These highly negative free energy values indicate a strong theoretical basis for spontaneous and stable ligand-target engagement. Additionally, we identified the top 8 “hot-spot” residues that contributed most significantly to the total binding free energy for each complex (**Supplementary Figure 2**). For instance, in the AKT1-Kaempferol complex, residues such as VAL270, LEU210, and GLN79 provided major energy contributions, primarily driven by favorable van der Waals forces and non-polar interactions that overcame unfavorable polar solvation energies. Similar robust energy contribution profiles from specific key residues (e.g., TYR, MET, and VAL variants) were observed stabilizing the quercetin complexes. In conclusion, computational simulations provide physicochemical evidence supporting the prediction that quercetin and kaempferol can directly target and bind to AKT1, PTGS2, and HSP90AA1 (**Figure 6**).

### 3.8 *In vivo* behavioral validation of ginseng-mulberry intervention on Cd-induced anxiety

**Figure 7:**
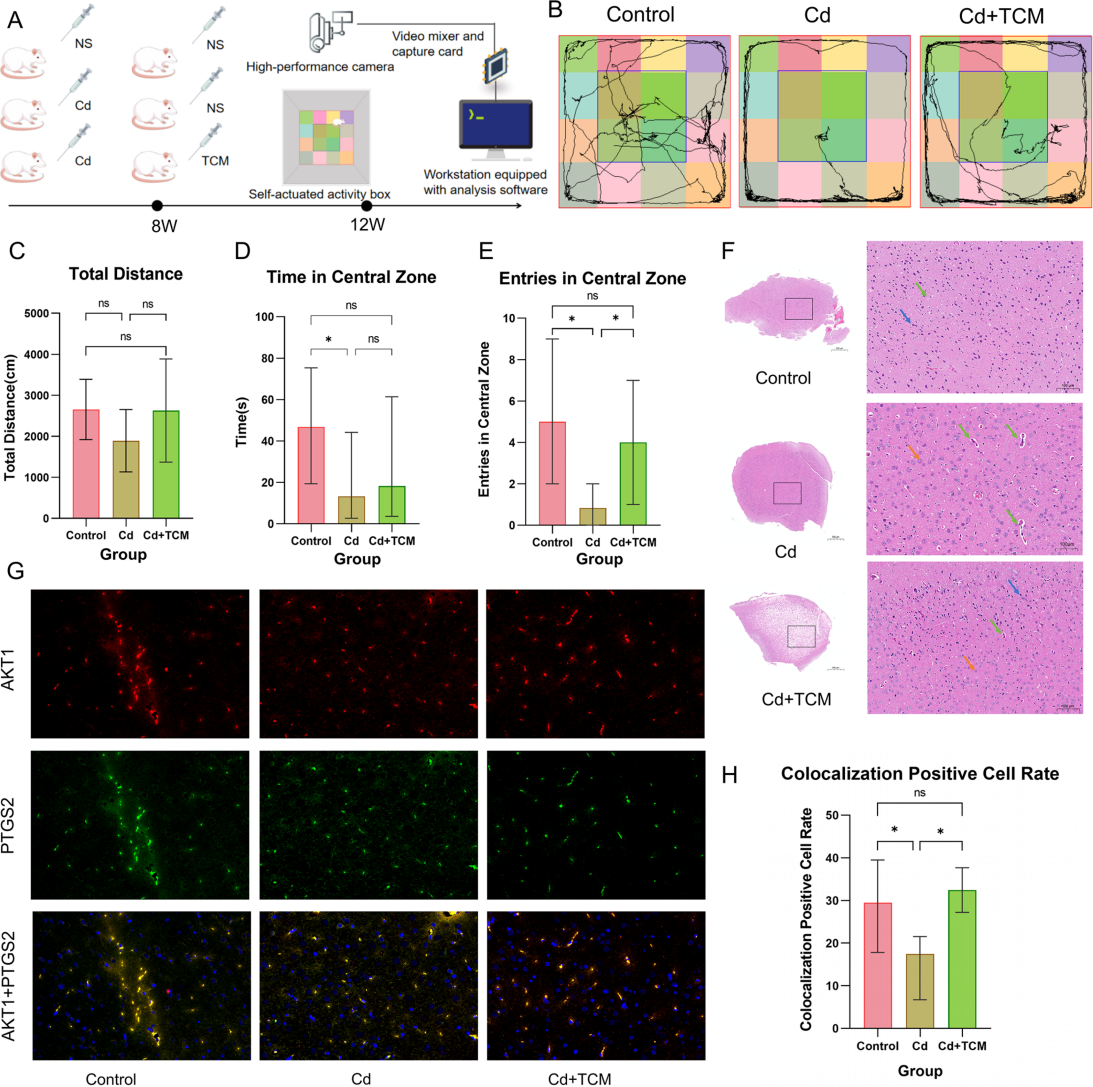
Establishment of the rat cadmium exposure model, behavioral testing. **(A)** Experimental design which including modeling and OFT. **(B)** Movement trajectory diagrams of rats in the open field test across three groups. **(C)** Comparison chart of total movement distance in rats. **(D)** Comparative chart of exploration time in the central zone of rats. **(E)** Comparison chart of entry counts in the central zone of rats. **(F)** Comparison of hematoxylin and eosin (HE) staining in the rat prefrontal cortex. The green arrow represents vascular spaces, the blue arrow represents normal glial cells, and the orange arrow represents glial cells that have become rounded. **(G)** Immunofluorescence analysis of AKT1 and PTGS2 expression in the rat prefrontal cortex. **(H)** Quantitative comparison of AKT1 and PTGS2 immunofluorescence co-localization positive cells in the rat prefrontal cortex. *P < 0.05. ns, not significant.

The schematic diagram of the experimental design is shown in **Figure 7A**, and representative OFT trajectories are shown in **Figure 7B**. Total distance traveled did not differ among groups (**Figure 7C**), suggesting intact baseline locomotion. Compared with controls, Cd-exposed rats showed fewer center entries and shorter center duration (P < 0.05), consistent with increased anxiety-like behavior. The ginseng-mulberry leaf decoction reversed these behavioral changes (**Figures 7D, E**). HE staining of the prefrontal cortex showed enlarged perivascular spaces and altered glial morphology in Cd-exposed rats, which were attenuated in the intervention group (**Figure 7F**). Based on network and docking results, we selected AKT1 and PTGS2 for *in vivo* validation. Immunofluorescence suggested Cd exposure reduced AKT1 and PTGS2 signals in the prefrontal cortex, while decoction treatment partially restored them (**Figures 7G, H**). Together, these results support a protective effect of the ginseng–mulberry leaf pair against Cd-associated anxiety-like behavior and prefrontal pathology.

## 4 Discussion

Cadmium (Cd) is a persistent environmental pollutant with a long biological half-life. It can perturb the central nervous system through oxidative stress and mitochondrial dysfunction, blood-brain barrier disruption, ionic mimicry, neurotransmitter imbalance, and regulated cell-death pathways (24–26). Current pharmacological interventions for anxiety disorders primarily include benzodiazepines and selective serotonin reuptake inhibitors (SSRIs) (27, 28). These drugs primarily act on neurotransmitter systems but can be limited by adverse effects and incomplete response (29, 30). These features motivate multi-target strategies that can address toxin-driven neuroimmune and metabolic dysregulation. Here, we investigated a medicine–food homologous ginseng-mulberry leaf pair as a candidate intervention.

Ginseng and mulberry leaf were prioritized by proteomics-seeded reverse screening and were further supported by traditional use records. This pairing aligns with a “tonifying and clearing” formulation logic and offers a pragmatic strategy for long-term use in exposure settings. At the molecular level, docking and molecular dynamics highlighted quercetin and kaempferol as key interactors, with stable binding to AKT1, PTGS2, and HSP90AA1. These computational results nominate plausible chemical drivers for downstream biological validation.

Mechanistically, ginsenoside Rg1 promotes neuronal regeneration and rescues synaptic plasticity by upregulating the hippocampal BDNF/TrkB signaling pathway (31). Conversely, mulberry leaf extract exerts neuroprotective effects by enhancing GABAergic transmission to antagonize heavy metal-induced glutamate excitotoxicity (32). Our enrichment analysis provided consistent results, revealing that the core targets were significantly enriched in the “pathways of neurodegeneration” and key neurotransmitter systems, including “serotonergic synapse” and “dopaminergic synapse”. Furthermore, our functional enrichment analysis highlights the important axis of oxidoreductase activity and xenobiotic response pathways. Toxicological models have demonstrated that cadmium exposure directly triggers ROS generation, which subsequently leads to dysregulated Akt signaling and strongly upregulates the expression of pro-inflammatory PTGS2 (COX-2). Although we did not directly quantify biochemical oxidative markers (e.g., ROS, MDA, or SOD) in the current study, substantial literature evidence supports the association between our predicted targets and cadmium-induced oxidative stress (33–37). Therefore, the effects on oxidative stress and anxiety hold great potential for further in-depth research.

At the molecular level, the biological functions of HSP90AA1, AKT1, and PTGS2 are closely associated with the pathological mechanisms of anxiety and neurotoxicity. Since our *in vivo* validation primarily focused on the total protein expression levels of the predicted targets as a preliminary screening effort, we rely on established literature to elucidate their roles in broader signaling cascades. These core targets collectively constitute the heart of the “neuro-immuno-metabolic axis”, functioning across three dimensions: maintaining neural structural stability, modulating cellular metabolic function, and monitoring the immune microenvironment. As an important molecular chaperone in neurons, HSP90AA1 not only maintains neurotransmitter release at presynaptic terminals but has also been shown to participate in the differentiation and migration of neural stem cells. Dysregulation of its function is often implicated in central nervous system injury and the progression of neurological disorders such as epilepsy (38, 39). AKT1, a central kinase of the PI3K/AKT signaling pathway, has been further highlighted in recent studies for its key role in antidepressant and anxiolytic effects: activation of AKT1 can significantly enhance spine density and synaptic plasticity in the prefrontal cortex and hippocampus via upregulation of the BDNF/mTOR signaling axis, thereby reversing stress-induced neuronal damage (40). In contrast, PTGS2 acts as a critical driver of the neuroinflammatory cascade. A systematic review noted that selective inhibition of COX-2 significantly reduces gliosis markers and levels of inflammatory cytokines, ultimately improving anxiety-like behaviors in chronic stress models (41, 42).

The prefrontal cortex is a key node for anxiety-related circuitry (43), and Cd exposure can damage neurovascular units and glial homeostasis (44). In our rat model, Cd reduced open-field center exploration and was accompanied by enlarged perivascular spaces and glial dysmorphology. The decoction improved both behavioral and histological readouts and was associated with restoration of AKT1 and PTGS2 immunofluorescence in the prefrontal cortex. While PTGS2 is canonically regarded as an inducible marker of neuroinflammation, its constitutive expression within the central nervous system is indispensable for the maintenance of neuronal synaptic plasticity (42, 45). Our co-localization results indicate that in the context of chronic cadmium neurotoxicity, the downregulation of PTGS2 does not signify a resolution of inflammatory responses; rather, it reflects an impairment of the AKT1-mediated neuronal survival and plasticity signaling pathway. Cadmium likely inhibits AKT1 phosphorylation, thereby disrupting the maintenance of downstream constitutive PTGS2 levels, which ultimately precipitates synaptic dysfunction and the onset of anxiety-like behaviors. Consequently, the reversal of PTGS2 expression by the ginseng-mulberry intervention fundamentally represents a restoration of compromised neuronal networks, rather than a mere anti-inflammatory effect.

This study has several limitations. First, the clinical proteomics analysis is based on a modest, cross-sectional matched cohort (n=50). Therefore, the identified proteomic signatures should be considered exploratory, warranting future external validation through longitudinal studies to assess their clinical translatability. Second, the material basis and computational predictions require further empirical confirmation. Without biochemical assays (e.g., SPR, LC-MS/MS), the MD simulations serve as hypothesis-generating structural models demonstrating physicochemical feasibility rather than definitive functional engagement. Finally, Our study lacks causal perturbation validation, and behavioral phenotyping was limited to a single paradigm (open field test) and brain region (prefrontal cortex). Therefore, AKT1, PTGS2, and HSP90AA1 are considered predictive hubs and exploratory mechanistic candidates rather than confirmed causal drivers.

Despite these limitations, the work contributes a clinical proteome-initiated discovery (CP-ID) workflow that starts from human exposure biology and progresses to convergent computational and *in vivo* validation. This “computational-to-biological” integrative approach is increasingly driving innovations across various medical domains. For instance, the integration of artificial intelligence with advanced imaging technologies has significantly enhanced the diagnostic and management pipelines for neurodegenerative diseases. Within the realm of neuropsychiatric phytomedicine, machine-learning-assisted network pharmacology strategies have been successfully employed to identify the precise molecular targets of herbal interventions, such as Hypericum perforatum for major depressive disorder. Similarly, our CP-ID framework provides a pragmatic and exploratory strategy, illustrating how integrating clinical omics with computational screening can effectively prioritize therapeutic candidates for complex environmental toxicities.

## Conclusion

Using a propensity-score-matched cadmium-exposed cohort, we identified a 120-protein plasma signature associated with anxiety. Proteomics-seeded network pharmacology prioritized a ginseng-mulberry leaf (medicine-food homologous) pair and highlighted AKT1/PTGS2/HSP90AA1 as predicted hub nodes. In Cd-exposed rats, the decoction improved open-field anxiety-like behavior and attenuated prefrontal neurovascular and glial pathology, with restoration of AKT1 and PTGS2 immunofluorescence. Collectively, these data support the CP-ID strategy as a data-driven route to nominate multi-target interventions for environmental toxin-associated neurobehavioral outcomes.

## Data Availability

The original contributions presented in the study are included in the article/**Supplementary Material**. Further inquiries can be directed to the corresponding author/s.
