# Supplementary material for: Ginseng-mulberry (medicine-food homologous) pair mitigates cadmium-induced anxiety: a clinical proteomics-guided network pharmacology with rat validation
Source: Front Psychiatry. 2026 May 25;17:1792233. doi: 10.3389/fpsyt.2026.1792233 (PMC13243265; doi:10.3389/fpsyt.2026.1792233)
Supplement: Supplementary file 1 [file Table1.docx]

Supplementary Methodology

1 Inclusion and Exclusion Criteria for Study Subjects.

1.1 Inclusion criteria for the case group (anxiety group).

(1) Participants are aged 45 and above. (2) Anxiety status assessment is conducted using the 14-item Hamilton Anxiety Rating Scale (HAMA-14). (3) Based on HAMA scores, classifications are as follows: a total score of ≥29 indicates possible severe anxiety; ≥21 indicates definite significant anxiety; ≥14 indicates definite anxiety; and a score exceeding 7 suggests possible anxiety. Consent to participate: Participants or their legal guardians must agree to participate in this study and sign an informed consent form. (4) Participants must not have used anti-anxiety or antipsychotic medications for at least 3 months. (5) Language comprehension: Participants must possess basic language comprehension and expression abilities to undergo testing and communication. (6) Residential environment: Participants are permanent residents of the cadmium-contaminated area in Gangkou Town, Jiujiang City, Jiangxi Province.

1.2 Inclusion criteria for the control group (non-anxiety group).

(1) Age: Participants must be 45 years old or above. (2) Anxiety Assessment: According to the HAMA score classification, participants with a score of less than 7 are considered to have no anxiety symptoms. (3) Consent to Participate: Participants or their legal guardians must agree to participate in this study and sign an informed consent form. (4) Language Comprehension: Participants must possess basic language comprehension and expression abilities, enabling them to complete tests and communicate effectively. (5) Residence: Participants must also be permanent residents of the cadmium-contaminated area in Gangkou Town, Jiujiang City, Jiangxi Province.

1.3 Exclusion Criteria.

(1) Age: Individuals under 45 years old are excluded. (2) Health status: Individuals with severe heart disease, lung disease, kidney disease, or other serious physical illnesses, as well as those with mental illnesses such as schizophrenia or severe depression, will be excluded. (3) Informed consent: Individuals who cannot provide their own consent or that of their legal guardian will be excluded. (4) Language comprehension: Individuals with severely impaired language comprehension and expression abilities, who cannot communicate effectively, will be excluded.

2 Quality Control.

To mitigate the risk of bias in the research process, this study implemented systematic controls for selection bias, information bias, and confounding bias.

2.1 Control of Selection Bias.

(1) Propensity Score Matching: A 1:1 matching was performed based on gender, age, BMI, and educational level to ensure comparability between the study and control groups on baseline variables. Gender was matched exactly, while differences in age and BMI were controlled to ≤ ±10%, and differences in educational level were controlled to ≤ ±3 years. Propensity scores were calculated using logistic regression, and the caliper method (±0.02 standard deviations) was applied to control matching precision, thereby reducing bias caused by non-random allocation.

(2) Strict Inclusion and Exclusion Criteria: Both the case and control groups were restricted to long-term residents of the cadmium-contaminated area in Gangkou Town, Jiujiang City, Jiangxi Province. Individuals aged <45 years or with severe physical or mental illnesses were excluded to minimize heterogeneity and potential confounding factors. Through population mobility screening (e.g., community registration), the risk of non-random attrition was minimized to ensure the sample represented long-term residents exposed to the contaminated environment.

2.2 Control of Information Bias

(1) Standardized Assessment Tools: Anxiety status was assessed using the Hamilton Anxiety Scale (HAMA14), which has been validated for reliability and validity in prior studies. Investigators were uniformly trained and passed a consistency assessment (Kappa value ≥ 0.85) to eliminate differences in subjective judgment.

(2) Blinding and Data Verification: During data collection, blinding was implemented for the anxiety grouping information of study subjects to prevent assessors from knowing the case/control status. Data were independently entered by two individuals, and accuracy was ensured through logical verification (e.g., range checking, retesting of outliers).

(3) Control of Confounding Information: Through medical record reviews and face-to-face interviews, individuals who recently used anxiolytics or antipsychotics were explicitly excluded to reduce the potential impact of medication interference on anxiety scores.

2.3 Control of Confounding Bias

(1) Stratified Matching and Covariate Adjustment: In addition to matching variables, potential confounding factors (e.g., occupation type, smoking history, duration of cadmium exposure) were further adjusted during statistical analysis. Multivariable Cox regression or generalized linear models were used to quantify the strength of associations.

(2) Doubly Robust Estimation with Combined Correction: Based on propensity score matching (PSM), non-parametric local regression (LOESS) smoothing was applied to covariates (cumulative cadmium exposure, annual household income) to construct a doubly robust estimation model for the exposure-anxiety association, enhancing resistance to bias in nonlinear effects of high-dose exposure.

3 Compliance with STROBE Guidelines.

This study adhered to the STROBE Statement throughout, standardizing the reporting of observational epidemiological research. This included: a clear description of the subject recruitment process (inclusion/exclusion criteria, matching methods) (with a flowchart attached). Detailed reporting of covariate definitions, data collection methods, and statistical model parameters. Potential bias sources (e.g., unmeasured confounders) and control measures were disclosed to enhance result interpretability and reproducibility.
