# Supplementary material for: Ginseng-mulberry (medicine-food homologous) pair mitigates cadmium-induced anxiety: a clinical proteomics-guided network pharmacology with rat validation
Source: Front Psychiatry. 2026 May 25;17:1792233. doi: 10.3389/fpsyt.2026.1792233 (PMC13243265; doi:10.3389/fpsyt.2026.1792233)
Supplement: Supplementary file 2 [file Table2.docx]

**Supplementary Figure 1 The network diagram of the compounds in ginseng and mulberry leaves with various targets.**


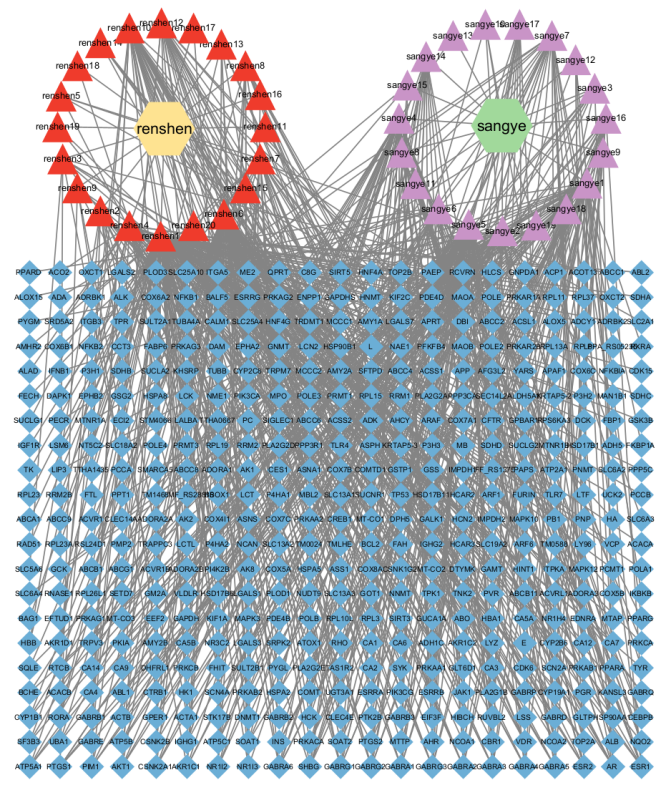


**Supplementary Figure 2 Binding free energy calculated by MM-PSA and surface potential distribution.**

**
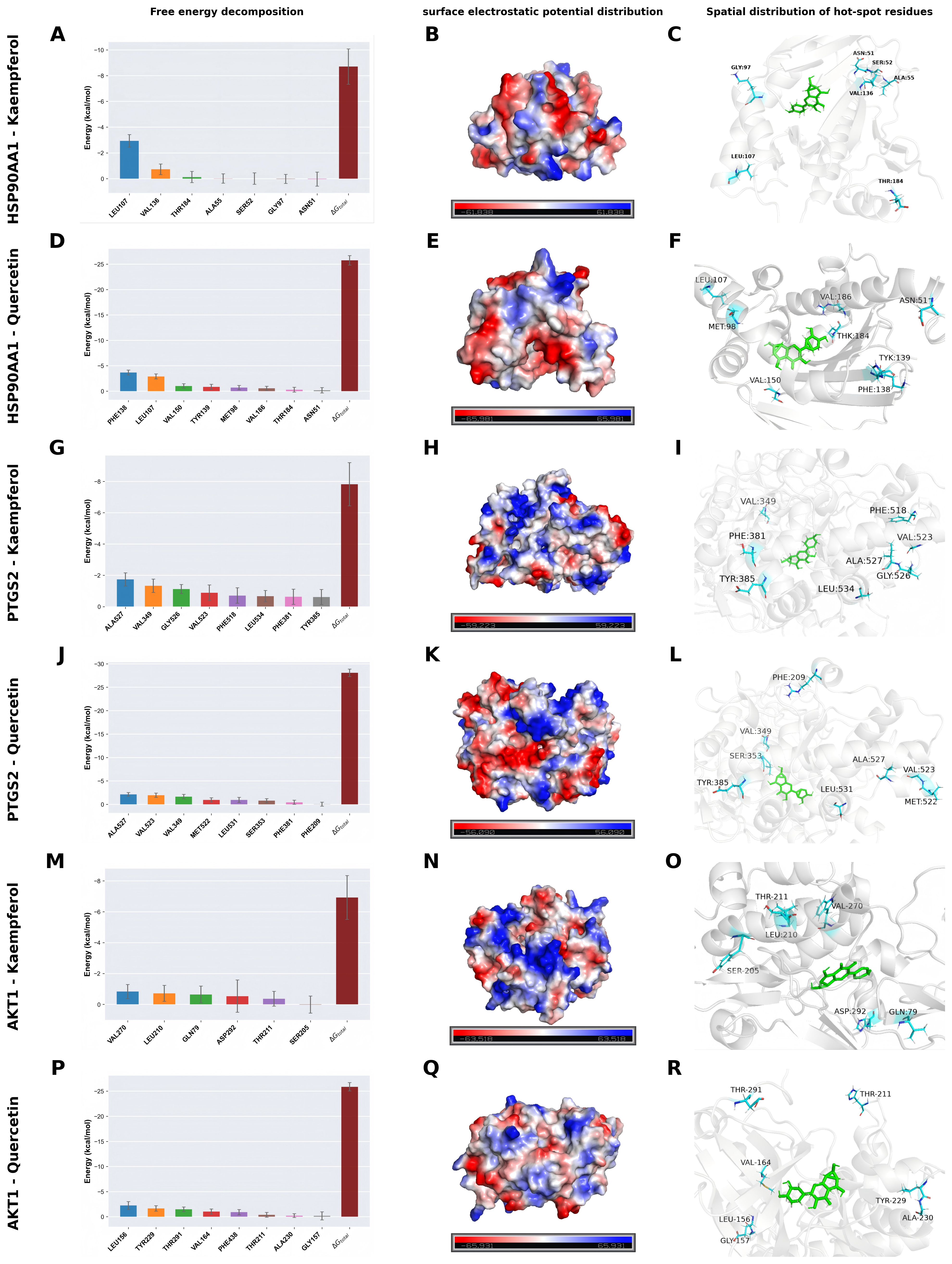
**
