# Supplementary material for: Ginseng-mulberry (medicine-food homologous) pair mitigates cadmium-induced anxiety: a clinical proteomics-guided network pharmacology with rat validation
Source: Front Psychiatry. 2026 May 25;17:1792233. doi: 10.3389/fpsyt.2026.1792233 (PMC13243265; doi:10.3389/fpsyt.2026.1792233)
Supplement: Supplementary file 3 [file Table3.docx]

**Supplementary Table 1 Baseline characteristics of the included population.**

| Anxiety | 0 | 1 | Standardize diff. | P-value |
| --- | --- | --- | --- | --- |
| N | 25 | 25 |  |  |
| Age (years, mean ± sd) | 62.92 ± 5.38 | 62.28 ± 6.79 | 0.10 (-0.45, 0.66) | 0.713 |
| Education (years, mean ± sd) | 11.60 ± 2.68 | 12.24 ± 2.91 | 0.23 (-0.33, 0.79) | 0.422 |
| BMI (kg/m2, mean ± sd) | 24.12 ± 2.63 | 23.83 ± 2.94 | 0.11 (-0.45, 0.66) | 0.711 |
| HAMD (mean ± sd) | 5.40 ± 5.97 | 11.96 ± 6.11 | 1.09 (0.49, 1.68) | <0.001 |
| HAMA (mean ± sd) | 3.28 ± 1.93 | 11.24 ± 3.84 | 2.62 (1.86, 3.37) | <0.001 |
| MMSE (mean ± sd) | 25.24 ± 3.37 | 22.68 ± 3.69 | 0.72 (0.15, 1.30) | 0.014 |
| VRF (mean ± sd) | 8.84 ± 3.51 | 9.80 ± 4.17 | 0.25 (-0.31, 0.81) | 0.383 |
| IADL (mean ± sd) | 14.88 ± 7.52 | 17.76 ± 9.68 | 0.33 (-0.23, 0.89) | 0.246 |
| VFT (mean ± sd) | 33.60 ± 10.28 | 28.12 ± 9.02 | 0.57 (0.00, 1.13) | 0.051 |
| TMT_A (s, mean ± sd) | 59.70 ± 21.61 | 83.80 ± 46.81 | 0.66 (0.09, 1.23) | 0.024 |
| TMT_B (s, mean ± sd) | 134.70 ± 89.43 | 142.42 ± 89.70 | 0.09 (-0.47, 0.64) | 0.762 |
| STROOP_WORD (s, mean ± sd) | 24.30 ± 8.78 | 36.03 ± 34.32 | 0.47 (-0.09, 1.03) | 0.104 |
| STROOP_COLOR (s, mean ± sd) | 30.50 ± 17.45 | 42.75 ± 37.35 | 0.42 (-0.14, 0.98) | 0.144 |
| STROOP_COLOR_WORD (s, mean ± sd) | 52.36 ± 35.47 | 63.50 ± 44.37 | 0.28 (-0.28, 0.83) | 0.332 |
| VST (s,mean ± sd) | 25.01 ± 27.37 | 24.15 ± 26.29 | 0.03 (-0.52, 0.59) | 0.910 |
| GLU (mmol/L, mean ± sd) | 6.13 ± 3.06 | 5.42 ± 1.44 | 0.30 (-0.26, 0.85) | 0.300 |
| TC (mmol/L, mean ± sd) | 4.03 ± 1.21 | 4.23 ± 1.04 | 0.17 (-0.38, 0.73) | 0.545 |
| TG (mmol/L, mean ± sd) | 1.59 ± 0.85 | 1.50 ± 0.68 | 0.11 (-0.45, 0.66) | 0.704 |
| LDL_C (mmol/L, mean ± sd) | 2.29 ± 0.87 | 2.48 ± 0.73 | 0.25 (-0.31, 0.80) | 0.389 |
| HDL_C (mmol/L, mean ± sd) | 1.10 ± 0.63 | 1.09 ± 0.30 | 0.01 (-0.54, 0.56) | 0.973 |
| Hg (μg/L, mean ± sd) | 3.80 ± 1.84 | 4.29 ± 1.85 | 0.27 (-0.29, 0.82) | 0.350 |
| Pb (μg/dL, mean ± sd) | 6.24 ± 3.00 | 4.59 ± 2.43 | 0.60 (0.04, 1.17) | 0.038 |
| Cd (μg/L, mean ± sd) | 3.69 ± 2.79 | 6.09 ± 3.02 | 0.82 (0.25, 1.40) | 0.005 |
| Age(≥65years) |  |  | 0.16 (-0.39, 0.72) | 0.564 |
| 0 | 14 (56.00%) | 16 (64.00%) |  |  |
| 1 | 11 (44.00%) | 9 (36.00%) |  |  |
| Gender (n, %) |  |  | 0.33 (-0.23, 0.88) | 0.254 |
| 0 | 12 (48.00%) | 16 (64.00%) |  |  |
| 1 | 13 (52.00%) | 9 (36.00%) |  |  |
| SMOKING (n, %) |  |  | 0.26 (-0.29, 0.82) | 0.355 |
| 0 | 19 (76.00%) | 16 (64.00%) |  |  |
| 1 | 6 (24.00%) | 9 (36.00%) |  |  |
| Hypertension (n, %) |  |  | 0.95 (0.36, 1.53) | 0.002 |
| 0 | 13 (52.00%) | 3 (12.00%) |  |  |
| 1 | 12 (48.00%) | 22 (88.00%) |  |  |
| DM (n, %) |  |  | 0.09 (-0.47, 0.64) | 0.758 |
| 0 | 17 (68.00%) | 18 (72.00%) |  |  |
| 1 | 8 (32.00%) | 7 (28.00%) |  |  |
| Hyperlipidemia (n, %) |  |  | 0.43 (-0.13, 0.99) | 0.136 |
| 0 | 14 (56.00%) | 19 (76.00%) |  |  |
| 1 | 11 (44.00%) | 6 (24.00%) |  |  |
| APOE4 (n, %) |  |  | 0.51 (-0.06, 1.07) | 0.083 |
| 0 | 13 (52.00%) | 7 (28.00%) |  |  |
| 1 | 12 (48.00%) | 18 (72.00%) |  |  |
| APOE3 (n, %) |  |  | 0.59 (0.02, 1.15) | 0.047 |
| 0 | 8 (32.00%) | 15 (60.00%) |  |  |
| 1 | 17 (68.00%) | 10 (40.00%) |  |  |
| HAMD (n, %) |  |  | 1.67 (1.02, 2.31) | <0.001 |
| 0 | 20 (80.00%) | 4 (16.00%) |  |  |
| 1 | 5 (20.00%) | 21 (84.00%) |  |  |
| MMSE (n, %) |  |  | 1.22 (0.61, 1.82) | <0.001 |
| 0 | 19 (76.00%) | 6 (24.00%) |  |  |
| 1 | 6 (24.00%) | 19 (76.00%) |  |  |
| Hg (>5.8μg/L) |  |  | 0.32 (-0.24, 0.87) | 0.269 |
| 0 | 22 (88.00%) | 19 (76.00%) |  |  |
| 1 | 3 (12.00%) | 6 (24.00%) |  |  |
| Pb ((>5μg/dL) |  |  | 0.59 (0.02, 1.15) | 0.047 |
| 0 | 8 (32.00%) | 15 (60.00%) |  |  |
| 1 | 17 (68.00%) | 10 (40.00%) |  |  |
| Cd (＞5μg/L) |  |  | 0.68 (0.11, 1.25) | 0.023 |
| 0 | 18 (72.00%) | 10 (40.00%) |  |  |
| 1 | 7 (28.00%) | 15 (60.00%) |  |  |

P-value*: For continuous variables, the P-value was calculated using the Kruskal-Wallis rank sum test. For count variables with a theoretical frequency of less than 10, Fisher's exact test was used to determine the P-value. "0" represents the non-anxiety group, and "1" represents the anxiety group.

**Supplementary Table 2 Correspondence Between Traditional Chinese Medicine (TCM) and Components.**

| TCM | Components |
| --- | --- |
| renshen1 | Adenosine,Adenine Nucleoside |
| renshen2 | Raffinose |
| renshen3 | Biotin |
| renshen4 | Succinic Acid |
| renshen5 | Octanal |
| renshen6 | Kaempferol |
| renshen7 | Panaxatriol |
| renshen8 | Neointermedeol |
| renshen9 | 3,4-Dihydroxybenzaldehyde,Hydroxybenzoic Acid,M-Hydroxybenzoic Acid,P-Hydroxybenzoic Acid,Salicylic Acid |
| renshen10 | Ginsenol |
| renshen11 | Panaxadiol |
| renshen12 | 2,6-Ditertbutyl-4-Methyl Phenol |
| renshen13 | Widdrol |
| renshen14 | Niacin,Nicotinic Acid |
| renshen15 | Cedrol,Eudesmol,Î‘-Cedrol |
| renshen16 | Panasinsanol A |
| renshen17 | Panasinsanol B |
| renshen18 | 2,5-Dimethyl-7-Hydroxy Chromone |
| renshen19 | Vitamin B1 |
| renshen20 | Deoxygomisin A |
| sangye1 | Pentanic Acid,Scopolin |
| sangye2 | Benzyl Alcohol O-Î’-D-Glucopyranoside,Campesterol |
| sangye3 | Adenine |
| sangye4 | 2',4'-Dihydroxy-7-Methoxy-8-Prenylflavan,5,7-Dihydroxychromone |
| sangye5 | Quercetin |
| sangye6 | Cudranin |
| sangye7 | Methyl Salicylate,Moracin C |
| sangye8 | Eugenol,Guaiacol |
| sangye9 | P-Cresol |
| sangye10 | Cudranin,M-Cresol |
| sangye11 | 5-Hydroxycoumarin,Guaiacol |
| sangye12 | Paeonol,Scopoletin |
| sangye13 | Butanoic Acid,Butyric Acid |
| sangye14 | Pavilion,Scopoletin,Scopoletol,Trigonelline |
| sangye15 | Fumaric Acid |
| sangye16 | O-Cresol |
| sangye17 | Moracin E |
| sangye18 | Morin |
| sangye19 | Hemolysin |

**Supplementary Table 3 Molecular Functions of 90 Targets**

| Gene | UniProt ID | Protein name | Molecular function | Reports related to anxiety [PubMed(gene name)AND(anxiety)] |
| --- | --- | --- | --- | --- |
| ABCB1 | P08183 | ATP-dependent translocase ABCB1 (P-glycoprotein 1) | Energy-dependent efflux pump responsible for decreased drug accumulation in multidrug-resistant cells(PubMed:2897240). | ABCB1 is associated with Anxiety [PMID: 36628952]. |
| ABCC1 | P33527 | Multidrug resistance-associated protein 1 (MRP1) | Mediates ATP-dependent, GSH-independent cyclic GMP-AMP (cGAMP) export . Thus, by limiting intracellular cGAMP concentrations negatively regulates the cGAS-STING pathway (PubMed:36070769). | ABCB1 is associated with Anxiety [PMID: 25991605]. |
| ABCC2 | Q92887 | Canalicular multispecific organic anion transporter 1 (MRP2) | ATP-dependent transporter of the ATP-binding cassette (ABC) family that binds and hydrolyzes ATP to enable active transport of various substrates including many drugs, toxicants and endogenous compound across cell membranes. Transports a wide variety of conjugated organic anions such as sulfate-, glucuronide- and glutathione (GSH)-conjugates of endo- and xenobiotics substrates (PubMed:16332456). | ABCC2 is associated with Anxiety [PMID: 36628952]. |
| ABCC4 | O15439 | Multidrug resistance-associated protein 4 (MRP4) | ATP-dependent transporter of the ATP-binding cassette (ABC) family that actively extrudes physiological compounds and xenobiotics from cells. Transports a range of endogenous molecules that have a key role in cellular communication and signaling, including cyclic nucleotides such as cyclic AMP (cAMP) and cyclic GMP (cGMP), bile acids, steroid conjugates, urate, and prostaglandins (PubMed:26721430). | None |
| ABCC6 | O95255 | Multidrug resistance-associated protein 6 (MRP6) | Inhibits TNF-alpha-mediated apoptosis through blocking one or more caspases.(PMID: 23912081) | None |
| ABCC8 | Q09428 | ATP-binding cassette sub-family C member 8 (SUR1) | KATP channels are regulated by cytoplasmic ATP/ADP ratios; ATP inhibits the channel by closing the pore, while ADP activates the channel ( PubMed:34815345). | None |
| ACP1 | P24666 | Low molecular weight phosphotyrosine protein phosphatase | Acts on tyrosine phosphorylated proteins, low-MW aryl phosphates and natural and synthetic acyl phosphates with differences in substrate specificity between isoform 1 and isoform 2.(PMID: 10336608) | None |
| ADA | P00813 | Adenosine deaminase | Acts as a positive modulator of adenosine receptors ADORA1 and ADORA2A, by enhancing their ligand affinity via conformational change (PubMed:23193172). | ADA is associated with Anxiety [PMID: 28074903] |
| ADORA1 | P30542 | Adenosine receptor A1 | Receptor for adenosine. The activity of this receptor is mediated by G proteins which inhibit adenylyl cyclase. | ADORA1 is associated with Anxiety [PMID: 33235193]. |
| ADORA2A | P29274 | Adenosine receptor A2a | Receptor for adenosine (By similarity). The activity of this receptor is mediated by G proteins which activate adenylyl cyclase (By similarity). | ADORA2 is associated with Anxiety [PMID: 33235193]. |
| AHCY | P23526 | Adenosylhomocysteinase | Catalyzes the hydrolysis of S-adenosyl-L-homocysteine to form adenosine and homocysteine (PubMed:10933798).Binds copper ions (By similarity). | None |
| AHR | P35869 | Aryl hydrocarbon receptor | Regulates a variety of biological processes, including angiogenesis, hematopoiesis, drug and lipid metabolism, cell motility and immune modulation (PubMed:12213388). | None |
| AKR1C1 | Q04828 | Aldo-keto reductase family 1 member C1 | Cytosolic aldo-keto reductase that catalyzes the NADH and NADPH-dependent reduction of ketosteroids to hydroxysteroids (PubMed:19218247). | AKR1C1 is associated with Anxiety [PMID: 24390875]. |
| AKR1C2 | P52895 | Aldo-keto reductase family 1 member C2 | Works in concert with the 5-alpha/5-beta-steroid reductases to convert steroid hormones into the 3-alpha/5-alpha and 3-alpha/5-beta-tetrahydrosteroids. Catalyzes the inactivation of the most potent androgen 5-alpha-dihydrotestosterone (5-alpha-DHT) to 5-alpha-androstane-3-alpha,17-beta-diol (3-alpha-diol) (PubMed:8573067). | AKR1C2 is associated with Anxiety [PMID: 37423029]. |
| AKT1 | P31749 | RAC-alpha serine/threonine-protein kinase (AKT1) | AKT plays a role as key modulator of the AKT-mTOR signaling pathway controlling the tempo of the process of newborn neurons integration during adult neurogenesis, including correct neuron positioning, dendritic development and synapse formation (By similarity). | AKT1 is associated with Anxiety [PMID: 33325370]. |
| ALAD | P13716 | Delta-aminolevulinic acid dehydratase | Catalyzes an early step in the biosynthesis of tetrapyrroles. Binds two molecules of 5-aminolevulinate per subunit, each at a distinct site, and catalyzes their condensation to form porphobilinogen. | ALAD is associated with Anxiety [PMID: 18639233]. |
| ALB | P02768 | Serum albumin | Binds to the bacterial siderophore enterobactin and inhibits enterobactin-mediated iron uptake of E.coli from ferric transferrin, and may thereby limit the utilization of iron and growth of enteric bacteria such as E.coli (PubMed:6234017). | None |
| ALOX15 | P16050 | Arachidonate 15-lipoxygenase | Finally, it is also involved in the cellular response to IL13/interleukin-13 (PubMed:21831839). | ALOX15 is associated with Anxiety [PMID: 29235036] |
| APAF1 | O14727 | Apoptotic protease-activating factor 1 | Oligomeric Apaf-1 mediates the cytochrome c-dependent autocatalytic activation of pro-caspase-9 (Apaf-3), leading to the activation of caspase-3 and apoptosis. This activation requires ATP. Isoform 6 is less effective in inducing apoptosis. | APAF1 is associated with Anxiety [PMID: 36041494] |
| APP | P05067 | Amyloid-beta precursor protein | Involved in cell mobility and transcription regulation through protein-protein interactions. Can promote transcription activation through binding to APBB1-KAT5 and inhibits Notch signaling through interaction with Numb. Couples to apoptosis-inducing pathways such as those mediated by G(o) and JIP. Inhibits G(o) alpha ATPase activity (By similarity). | APP is associated with Anxiety [PMID: 38572429] |
| AR | P10275 | Androgen receptor | Transcription factor activity is modulated by bound coactivator and corepressor proteins like ZBTB7A that recruits NCOR1 and NCOR2 to the androgen response elements/ARE on target genes, negatively regulating androgen receptor signaling and androgen-induced cell proliferation (PubMed:20812024). | None |
| ATOX1 | O00244 | Copper transport protein ATOX1 | Binds and deliver cytosolic copper to the copper ATPase proteins. May be important in cellular antioxidant defense. | None |
| BCHE | P06276 | Cholinesterase | Esterase with broad substrate specificity. Contributes to the inactivation of the neurotransmitter acetylcholine. Can degrade neurotoxic organophosphate esters. | BCHE is associated with Anxiety [PMID: 36628952] |
| BCL2 | P10415 | Apoptosis regulator Bcl-2 | May attenuate inflammation by impairing NLRP1-inflammasome activation, hence CASP1 activation and IL1B release (PubMed:17418785). | BCL2 is associated with Anxiety [PMID: 36747075] |
| CA1 | P00915 | Carbonic anhydrase 1 | Catalyzes the reversible hydration of carbon dioxide.Can hydrate cyanamide to urea (PubMed:10550681). | None |
| CA2 | P00918 | Carbonic anhydrase 2 | Catalyzes the reversible hydration of carbon dioxide (PubMed:9398308). | None |
| CA9 | Q16790 | Carbonic anhydrase 9 | Catalyzes the interconversion between carbon dioxide and water and the dissociated ions of carbonic acid (i.e. bicarbonate and hydrogen ions). | None |
| CFTR | P13569 | Cystic fibrosis transmembrane conductance regulator | Modulates the activity of the epithelial sodium channel (ENaC) complex, in part by regulating the cell surface expression of the ENaC complex(PubMed:27941075). | CFTR is associated with Anxiety [PMID: 37655981] |
| COMT | P21964 | Catechol-O-methyltransferase | Catalyzes the O-methylation, and thereby the inactivation, of catecholamine neurotransmitters and catechol hormones. Also shortens the biological half-lives of certain neuroactive drugs, like L-DOPA, alpha-methyl DOPA and isoproterenol. | COMT is associated with Anxiety [PMID: 36628952][PMID: 37101588] |
| CREB1 | P16220 | Cyclic AMP-responsive element-binding protein 1 | Regulates the expression of apoptotic and inflammatory response factors in cardiomyocytes in response to ERFE-mediated activation of AKT signaling (By similarity). | CREB1 is associated with Anxiety [PMID: 33017682] |
| CYP19A1 | P11511 | Aromatase (CYP19A1) | A cytochrome P450 monooxygenase that catalyzes the conversion of C19 androgens, androst-4-ene-3,17-dione (androstenedione) and testosterone to the C18 estrogens, estrone and estradiol, respectively (PubMed:2848247). | CYP19A1 is associated with Anxiety [PMID: 32450286] |
| CYP1B1 | Q16678 | Cytochrome P450 1B1 | A cytochrome P450 monooxygenase involved in the metabolism of various endogenous substrates, including fatty acids, steroid hormones and vitamins (PubMed:20972997). | CYP19A1 is associated with Anxiety [PMID: 37101588] |
| CYP2B6 | P20813 | Cytochrome P450 2B6 | A cytochrome P450 monooxygenase involved in the metabolism of endocannabinoids and steroids ( PubMed:21289075). | CYP2B6 is associated with Anxiety [PMID: 37032427] |
| CYP2C8 | P10632 | Cytochrome P450 2C8 | A cytochrome P450 monooxygenase involved in the metabolism of various endogenous substrates, including fatty acids, steroid hormones and vitamins ( PubMed:7574697). | None |
| DNMT1 | P26358 | DNA (cytosine-5)-methyltransferase 1 | Associates with DNA replication sites in S phase maintaining the methylation pattern in the newly synthesized strand, that is essential for epigenetic inheritance. (PubMed:24623306). | DNMT1 is associated with Anxiety [PMID: 24968059] |
| ENPP1 | P22413 | Ectonucleotide pyrophosphatase/phosphodiesterase family member 1 | Nucleotide pyrophosphatase that generates diphosphate (PPi) and functions in bone mineralization and soft tissue calcification by regulating pyrophosphate levels (By similarity). | None |
| ESR1 | P03372 | Estrogen receptor alpha | Maintains neuronal survival in response to ischemic reperfusion injury when in the presence of circulating estradiol (17-beta-estradiol/E2) (By similarity). | ESR1 is associated with Anxiety [PMID: 36386232][PMID: 31975979] |
| ESR2 | Q92731 | Estrogen receptor beta | Nuclear hormone receptor. Binds estrogens with an affinity similar to that of ESR1/ER-alpha, and activates expression of reporter genes containing estrogen response elements (ERE) in an estrogen-dependent manner (PubMed:20074560). | ESR2 is associated with Anxiety [PMID: 31975979] |
| FECH | P22830 | Ferrochelatase | Catalyzes the ferrous insertion into protoporphyrin IX and participates in the terminal step in the heme biosynthetic pathway. | None |
| FHIT | P49789 | Fragile histidine triad protein | Plays a role in the induction of apoptosis via SRC and AKT1 signaling pathways (PubMed:16407838). | FHIT is associated with Anxiety [PMID: 29249830] |
| FTL | P02792 | Ferritin light chain | Stores iron in a soluble, non-toxic, readily available form. Important for iron homeostasis. Iron is taken up in the ferrous form and deposited as ferric hydroxides after oxidation. Also plays a role in delivery of iron to cells. Mediates iron uptake in capsule cells of the developing kidney (By similarity). | FTL is associated with Anxiety [PMID: 33200454] |
| GABRB3 | P28472 | Gamma-aminobutyric acid receptor subunit beta-3 | GABAARs containing alpha-1 and beta-3 subunits exhibit synaptogenic activity; the gamma-2 subunit being necessary but not sufficient to induce rapid synaptic contacts formation (PubMed:25489750). | GABRB3 is associated with Anxiety [PMID: 27293187] |
| GAPDH | P04406 | Glyceraldehyde-3-phosphate dehydrogenase | Nuclear functions are probably due to the nitrosylase activity that mediates cysteine S-nitrosylation of nuclear target proteins such as SIRT1, HDAC2 and PRKDC (By similarity). | GAPDH is associated with Anxiety [PMID: 34603401] |
| GPER1 | Q99527 | G protein-coupled estrogen receptor 1 | Stimulates cAMP production, calcium mobilization and tyrosine kinase Src inducing the release of heparin-bound epidermal growth factor (HB-EGF) and subsequent transactivation of the epidermal growth factor receptor (EGFR), activating downstream signaling pathways such as PI3K/Akt and ERK/MAPK.Promotes neuritogenesis in developing hippocampal neurons. Plays a role in acute neuroprotection against NMDA-induced excitotoxic neuronal death. | GPER1 is associated with Anxiety [PMID: 28465157] |
| GSS | P48637 | Glutathione synthetase | Glutathione (gamma-glutamylcysteinylglycine, GSH) is the most abundant intracellular thiol in living aerobic cells and is required for numerous processes including the protection of cells against oxidative damage, amino acid transport, the detoxification of foreign compounds, the maintenance of protein sulfhydryl groups in a reduced state and acts as a cofactor for a number of enzymes (PubMed:10369661). | None |
| GSTP1 | P09211 | Glutathione S-transferase P | Negatively regulates CDK5 activity via p25/p35 translocation to prevent neurodegeneration | GSTP1 is associated with Anxiety [PMID: 36628952] |
| HBB | P68871 | Hemoglobin subunit beta | Functions as an endogenous inhibitor of enkephalin-degrading enzymes such as DPP3, and as a selective antagonist of the P2RX3 receptor which is involved in pain signaling, these properties implicate it as a regulator of pain and inflammation. | None |
| HCN2 | Q9UL51 | Potassium/sodium hyperpolarization-activated cyclic nucleotide-gated channel 2 | Contributes to the native pacemaker currents in heart (If) and in neurons (Ih) ( PubMed:10524219). Involved in the initiation of neuropathic pain in sensory neurons (By similarity). | HCN2 is associated with Anxiety [PMID: 39914775] |
| HSP90AA1 | P07900 | Heat shock protein HSP 90-alpha | Molecular chaperone that promotes the maturation, structural maintenance and proper regulation of specific target proteins involved for instance in cell cycle control and signal transduction. Binds bacterial lipopolysaccharide (LPS) and mediates LPS-induced inflammatory response, including TNF secretion by monocytes (PubMed:11276205). | HSP90AA1 is associated with Anxiety [PMID: 36624423] |
| HSP90B1 | P14625 | Heat shock protein HSP 90-beta (GRP94) | Molecular chaperone that functions in the processing and transport of secreted proteins (By similarity). | None |
| HSPA5 | P11021 | Heat shock 70 kDa protein 5 (BiP) | Endoplasmic reticulum chaperone that plays a key role in protein folding and quality control in the endoplasmic reticulum lumen ( PubMed:28332555). | HSPA5 is associated with Anxiety [PMID: 39567526] |
| HSPA8 | P11142 | Heat shock cognate 71 kDa protein | Molecular chaperone implicated in a wide variety of cellular processes, including protection of the proteome from stress, folding and transport of newly synthesized polypeptides, chaperone-mediated autophagy, activation of proteolysis of misfolded proteins, formation and dissociation of protein complexes, and antigen presentation. Plays a pivotal role in the protein quality control system, ensuring the correct folding of proteins, the re-folding of misfolded proteins and controlling the targeting of proteins for subsequent degradation (PubMed:36586411). | HSPA8 is associated with Anxiety [PMID: 26780340] |
| IKBKB | O14920 | Inhibitor of nuclear factor kappa-B kinase subunit beta | Serine kinase that plays an essential role in the NF-kappa-B signaling pathway which is activated by multiple stimuli such as inflammatory cytokines, bacterial or viral products, DNA damages or other cellular stresses ( PubMed:9346484). | None |
| LCK | P06239 | Tyrosine-protein kinase Lck | Non-receptor tyrosine-protein kinase that plays an essential role in the selection and maturation of developing T-cells in the thymus and in the function of mature T-cells. | None |
| LCN2 | P80188 | Neutrophil gelatinase-associated lipocalin | Iron-trafficking protein involved in multiple processes such as apoptosis, innate immunity and renal development ( PubMed:27780864). | LCN2 is associated with Anxiety [PMID: 38589429] |
| LTF | P02788 | Lactotransferrin | Transferrins are iron binding transport proteins which can bind two Fe3+ ions in association with the binding of an anion, usually bicarbonate. | None |
| MAPK10 | P53779 | Mitogen-activated protein kinase 10 (JNK3) | Serine/threonine-protein kinase involved in various processes such as neuronal proliferation, differentiation, migration and programmed cell death. | None |
| MAPK3 | Q16659 | Mitogen-activated protein kinase 3 (ERK1) | Atypical MAPK protein. Phosphorylates microtubule-associated protein 2 (MAP2) and MAPKAPK5. | MAPK3 is associated with Anxiety [PMID: 37968726] |
| MB | P02144 | Myoglobin | Monomeric heme protein which primary function is to store oxygen and facilitate its diffusion within muscle tissues. Reversibly binds oxygen through a pentacoordinated heme iron and enables its timely and efficient release as needed during periods of heightened demand ( PubMed:34679218). | None |
| MT-CO2 | P00403 | Cytochrome c oxidase subunit 2 | Component of the cytochrome c oxidase, the last enzyme in the mitochondrial electron transport chain which drives oxidative phosphorylation. | None |
| NFKB1 | P19838 | Nuclear factor NF-kappa-B p105 subunit | NF-kappa-B is a pleiotropic transcription factor present in almost all cell types and is the endpoint of a series of signal transduction events that are initiated by a vast array of stimuli related to many biological processes such as inflammation, immunity, differentiation, cell growth, tumorigenesis and apoptosis. | NFKB1 is associated with Anxiety [PMID: 20399847]. |
| P3H1 | Q32P28 | Prolyl 3-hydroxylase 1 (LEPRE1) | Basement membrane-associated chondroitin sulfate proteoglycan (CSPG). Has prolyl 3-hydroxylase activity catalyzing the post-translational formation of 3-hydroxyproline in -Xaa-Pro-Gly- sequences in collagens, especially types IV and V. May be involved in the secretory pathway of cells. Has growth suppressive activity in fibroblasts. | None |
| PCCB | P05166 | Propionyl-CoA carboxylase beta chain | This is one of the 2 subunits of the biotin-dependent propionyl-CoA carboxylase (PCC), a mitochondrial enzyme involved in the catabolism of odd chain fatty acids, branched-chain amino acids isoleucine, threonine, methionine, and valine and other metabolites ( PubMed:6765947). | None |
| PDE4D | Q08499 | cAMP-specific 3',5'-cyclic phosphodiesterase 4D | Hydrolyzes the second messenger cAMP, which is a key regulator of many important physiological processes. | PDE4D is associated with Anxiety [PMID: 17700644] |
| PGR | P06401 | Progesterone receptor | The steroid hormones and their receptors are involved in the regulation of eukaryotic gene expression and affect cellular proliferation and differentiation in target tissues. Depending on the isoform, progesterone receptor functions as a transcriptional activator or repressor. | None |
| PIK3CG | P48736 | Phosphatidylinositol 4,5-bisphosphate 3-kinase catalytic subunit gamma | Involved in immune, inflammatory and allergic responses. | None |
| POLE | Q07864 | DNA polymerase epsilon catalytic subunit | Catalytic component of the DNA polymerase epsilon complex (PubMed:10801849). | None |
| PPARA | Q07869 | Peroxisome proliferator-activated receptor alpha | Ligand-activated transcription factor. Key regulator of lipid metabolism. | PPARA is associated with Anxiety [PMID: 33312191] |
| PPARG | P37231 | Peroxisome proliferator-activated receptor gamma | Nuclear receptor that binds peroxisome proliferators such as hypolipidemic drugs and fatty acids. | PPARG is associated with Anxiety [PMID: 33312191] |
| PRKCA | P17252 | Protein kinase C alpha | Calcium-activated, phospholipid- and diacylglycerol (DAG)-dependent serine/threonine-protein kinase that is involved in positive and negative regulation of cell proliferation, apoptosis, differentiation, migration and adhesion, tumorigenesis, cardiac hypertrophy, angiogenesis, platelet function and inflammation, by directly phosphorylating targets such as RAF1, BCL2, CSPG4, TNNT2/CTNT, or activating signaling cascade involving MAPK1/3 (ERK1/2) and RAP1GAP. | PRKCA is associated with Anxiety [PMID: 39337965] |
| PTGS1 | P23219 | Prostaglandin G/H synthase 1 (COX-1) | Dual cyclooxygenase and peroxidase that plays an important role in the biosynthesis pathway of prostanoids, a class of C20 oxylipins mainly derived from arachidonate ((5Z,8Z,11Z,14Z)-eicosatetraenoate, AA, C20:4(n-6)), with a particular role in the inflammatory response. | PTGS1 is associated with Anxiety [PMID: 36386232] |
| PTGS2 | P35354 | Prostaglandin G/H synthase 2 (COX-2) | Dual cyclooxygenase and peroxidase in the biosynthesis pathway of prostanoids, a class of C20 oxylipins mainly derived from arachidonate ((5Z,8Z,11Z,14Z)-eicosatetraenoate, AA, C20:4(n-6)), with a particular role in the inflammatory response (PubMed:9261177). | PTGS2 is associated with Anxiety [PMID: 36104533]. |
| RCVRN | P35243 | Recoverin | Acts as a calcium sensor and regulates phototransduction of cone and rod photoreceptor cells (By similarity). | None |
| RNASE1 | P07998 | Ribonuclease pancreatic | Endonuclease that catalyzes the cleavage of RNA on the 3' side of pyrimidine nucleotides. Acts on single-stranded and double-stranded RNA. | None |
| RPL11 | P62913 | 60S ribosomal protein L11 | Component of the ribosome, a large ribonucleoprotein complex responsible for the synthesis of proteins in the cell (PubMed:32669547). | None |
| SDHD | O14521 | Succinate dehydrogenase [ubiquinone] cytochrome b small subunit | Membrane-anchoring subunit of succinate dehydrogenase (SDH) that is involved in complex II of the mitochondrial electron transport chain and is responsible for transferring electrons from succinate to ubiquinone (coenzyme Q) (PubMed:9533030). | SDHD is associated with Anxiety [PMID: 30536464] |
| SIRT3 | Q9NTG7 | NAD-dependent protein deacetylase sirtuin-3 | NAD-dependent protein deacetylase ( PubMed:24252090). | SIRT3 is associated with Anxiety [PMID: 31015456] |
| SLC13A1 | Q9BZW2 | Sodium-dependent dicarboxylate transporter | Sodium:sulfate symporter that mediates sulfate reabsorption in the kidney and small intestine (PubMed:11161786). | None |
| SLC2A1 | P11166 | Solute carrier family 2, facilitated glucose transporter member 1 (GLUT1) | Most important energy carrier of the brain: present at the blood-brain barrier and assures the energy-independent, facilitative transport of glucose into the brain (PubMed:10227690). | SLC2A1 is associated with Anxiety [PMID: 18780771] |
| SLC6A2 | P23975 | Sodium-dependent noradrenaline transporter (NET) | Mediates sodium- and chloride-dependent transport of norepinephrine (also known as noradrenaline) (PubMed:8125921). | SLC6A2 is associated with Anxiety [PMID: 36628952] |
| SLC6A3 | Q01959 | Sodium-dependent dopamine transporter (DAT) | Mediates sodium- and chloride-dependent transport of dopamine (PubMed:8302271). | SLC6A3 is associated with Anxiety [PMID: 36628952] |
| SLC6A4 | P31645 | Sodium-dependent serotonin transporter (SERT) | Essential for serotonin homeostasis in the central nervous system. In the developing somatosensory cortex, acts in glutamatergic neurons to control serotonin uptake and its trophic functions accounting for proper spatial organization of cortical neurons and elaboration of sensory circuits. In the mature cortex, acts primarily in brainstem raphe neurons to mediate serotonin uptake from the synaptic cleft back into the pre-synaptic terminal thus terminating serotonin signaling at the synapse (By similarity). | SLC6A4 is associated with Anxiety [PMID: 36628952] |
| TLR4 | O00206 | Toll-like receptor 4 | Transmembrane receptor that functions as a pattern recognition receptor recognizing pathogen- and damage-associated molecular patterns (PAMPs and DAMPs) to induce innate immune responses via downstream signaling pathways (PubMed:29038465). | TLR4 is associated with Anxiety [PMID: 30033154] |
| TLR7 | Q9NYK1 | Toll-like receptor 7 | Endosomal receptor that plays a key role in innate and adaptive immunity ( PubMed:32433612). | TLR7 is associated with Anxiety [PMID: 30610201] |
| TP53 | P04637 | Cellular tumor antigen p53 | Specifically methylates cytosineMultifunctional transcription factor that induces cell cycle arrest, DNA repair or apoptosis upon binding to its target DNA sequence (PubMed:9840937). | TP53 is associated with Anxiety [PMID: 38936518] |
| TRDMT1 | O14717 | tRNA (cytosine(38)-C(5))-methyltransferase | Specifically methylates cytosine 38 in the anticodon loop of tRNA(Asp) (PubMed:16424344). | None |
| TUBB | P07437 | Tubulin beta chain | Tubulin is the major constituent of microtubules, a cylinder consisting of laterally associated linear protofilaments composed of alpha- and beta-tubulin heterodimers. Microtubules grow by the addition of GTP-tubulin dimers to the microtubule end, where a stabilizing cap forms. Below the cap, tubulin dimers are in GDP-bound state, owing to GTPase activity of alpha-tubulin. | None |
| TYR | P14679 | Tyrosinase | This is a copper-containing oxidase that functions in the formation of pigments such as melanins and other polyphenolic compounds. Catalyzes the initial and rate limiting step in the cascade of reactions leading to melanin production from tyrosine (By similarity). | TYR is associated with Anxiety [PMID: 35884322] |
| VCP | P55072 | Transitional endoplasmic reticulum ATPase (Valosin-containing protein) | Necessary for the fragmentation of Golgi stacks during mitosis and for their reassembly after mitosis. | VCP is associated with Anxiety [PMID: 25716352] |
| VDR | P11473 | Vitamin D3 receptor | Nuclear receptor for calcitriol, the active form of vitamin D3 which mediates the action of this vitamin on cells (PubMed:37478846). | VDR is associated with Anxiety [PMID: 39065743]. |

**Supplementary Table 4 Previous studies on chemical characterization evidence for the predicted components.**

| **Herb Source** | **Predicted Compounds** | **Reference** | **Analytical Method / Evidence** |
| --- | --- | --- | --- |
| Ginseng | Ginsenosides; Quercetin | Li, K., Wang, Y. J., Chen, C., et al. (2025). Targeting pyroptosis: A novel strategy of ginseng for the treatment of diabetes and its chronic complications. Phytomedicine, 138, 156430. | It is clearly indicated that ginseng contains various active monomers, such as Ginsenoside Rg5, Rg3, Rb1, and Quercetin. The literature has reviewed the core roles of these components in regulating signaling pathways. |
|  | Kaempferol | Kim, T. H., Ku, S. K., Lee, I. C., & Bae, J. S. (2012). Anti-inflammatory effects of kaempferol-3-O-sophoroside in human endothelial cells. Inflammation Research, 61(3), 217-224. | Kaempferol-3-O-sophoroside (KPOS) was successfully isolated from ginseng leaves and confirmed to possess significant barrier-protective and anti-inflammatory activities. |
|  | Quercetin; Kaempferol | Kim, J. S. (2016). Investigation of phenolic, flavonoid, and vitamin contents in different parts of Korean Ginseng (Panax ginseng C.A. Meyer). Preventive Nutrition and Food Science, 21(3), 263. | Flavonoids were detected in different parts of ginseng (root and leaf) using GC/MS and HPLC-DAD, among which Quercetin and Kaempferol were identified as the main flavonoid components in the leaves. |
|  | Ginsenosides; Kaempferol | Wang, X., Kim, M., Han, R., et al. (2024). Increasing the amounts of bioactive components in American Ginseng (Panax quinquefolium L.) leaves using far-infrared irradiation. Foods, 13(4), 607. | Quantitative analysis using HPLC confirmed that heat treatment (e.g., far-infrared irradiation) significantly enhanced the contents of Ginsenoside Rd, total phenolics, and total flavonoids in ginseng leaves, demonstrating its enrichment in a large number of neuroprotective and antioxidant active substances. |
| Mulberry Leaf | Quercetin; Kaempferol | Lin, Z., Gan, T., Huang, Y., et al. (2022). Anti-inflammatory activity of mulberry leaf flavonoids in vitro and in vivo. International Journal of Molecular Sciences, 23(14), 7694. | The flavonoid components in mulberry leaves were detected using liquid chromatography-mass spectrometry (LC-MS), identified primarily as glycoside derivatives of quercetin and kaempferol. Furthermore, mulberry leaves were found to hold potential as a functional food or natural remedy for alleviating inflammatory diseases such as ulcerative colitis. |
|  | Kaempferol; Quercetin | Zheng, Q., Tan, W., Feng, X., et al. (2022). Protective effect of flavonoids from mulberry leaf on AAPH-induced oxidative damage in sheep erythrocytes. Molecules, 27(21), 7625. | Confirmed the presence of Quercetin and Kaempferol in Mulberry Leaf Flavonoids (MLF) via standard-based comparison and AlCl₃ colorimetric assays. The study validated that these specific flavonoids are responsible for potent antioxidant and cell-protective activities. |
